# Estimating pathogen‐spillover risk using host–ectoparasite interactions

**DOI:** 10.1002/ece3.11509

**Published:** 2024-06-18

**Authors:** Reilly N. Brennan, Sally L. Paulson, Luis E. Escobar

**Affiliations:** ^1^ Department of Entomology Virginia Tech Blacksburg Virginia USA; ^2^ Department of Fish and Wildlife Conservation Virginia Tech Blacksburg Virginia USA; ^3^ Center for Emerging, Zoonotic and Arthropod‐Borne Pathogens Virginia Tech Blacksburg Virginia USA; ^4^ Global Change Center Virginia Tech Blacksburg Virginia USA; ^5^ The Kellogg Center for Philosophy, Politics, and Economics Virginia Tech Blacksburg Virginia USA

**Keywords:** ectoparasites, hantavirus, network, rodent, spillover, wildlife, zoonotic

## Abstract

Pathogen spillover corresponds to the transmission of a pathogen or parasite from an original host species to a novel host species, preluding disease emergence. Understanding the interacting factors that lead to pathogen transmission in a zoonotic cycle could help identify novel hosts of pathogens and the patterns that lead to disease emergence. We hypothesize that ecological and biogeographic factors drive host encounters, infection susceptibility, and cross‐species spillover transmission. Using a rodent–ectoparasite system in the Neotropics, with shared ectoparasite associations as a proxy for ecological interaction between rodent species, we assessed relationships between rodents using geographic range, phylogenetic relatedness, and ectoparasite associations to determine the roles of generalist and specialist hosts in the transmission cycle of hantavirus. A total of 50 rodent species were ranked on their centrality in a network model based on ectoparasites sharing. Geographic proximity and phylogenetic relatedness were predictors for rodents to share ectoparasite species and were associated with shorter network path distance between rodents through shared ectoparasites. The rodent–ectoparasite network model successfully predicted independent data of seven known hantavirus hosts. The model predicted five novel rodent species as potential, unrecognized hantavirus hosts in South America. Findings suggest that ectoparasite data, geographic range, and phylogenetic relatedness of wildlife species could help predict novel hosts susceptible to infection and possible transmission of zoonotic pathogens. Hantavirus is a high‐consequence zoonotic pathogen with documented animal‐to‐animal, animal‐to‐human, and human‐to‐human transmission. Predictions of new rodent hosts can guide active epidemiological surveillance in specific areas and wildlife species to mitigate hantavirus spillover transmission risk from rodents to humans. This study supports the idea that ectoparasite relationships among rodents are a proxy of host species interactions and can inform transmission cycles of diverse pathogens circulating in wildlife disease systems, including wildlife viruses with epidemic potential, such as hantavirus.

## Graphical abstract

Understanding the interacting factors that lead to pathogen transmission in a zoonotic cycle could help identify novel hosts of pathogens and the patterns that lead to disease emergence. We use parasite ecology, phylogenetics, and geography to predict known and unknown hosts of hantavirus. This improves our knowledge of how host interaction influences disease transmission and can be used to inform targeted surveillance programs.

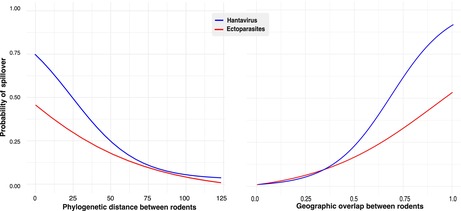

## 1 INTRODUCTION

Zoonotic diseases originate in animals and infect humans, posing significant threats to public health worldwide (Cutler et al., 2010; Holmes, 2022). Cross‐species spillover transmission, where a pathogen or parasite is transmitted from its reservoir host species (i.e., original or donor host) to a novel species (i.e., recipient host), is a critical precursor to zoonotic disease emergence (Kreuder Johnson et al., 2015; Olival et al., 2017). The possibility of pathogen spillover into novel species depends on many factors, some of which are directly associated with the reservoir host, including host distribution, density, and interactions with other potential hosts (Plowright et al., 2017). Understanding the underlying factors and mechanisms that drive pathogen transmission cycles in wildlife is crucial for identifying novel hosts and uncovering the ecological patterns that contribute to the emergence of diseases in humans (Alexander et al., 2018; Sánchez et al., 2021).

One group of zoonotic pathogens with global concern is hantaviruses. Hantaviruses are members of the family Hantaviridae and are primarily transmitted to humans through contact with infected rodents or their excreta (Laenen et al., 2019; Vial et al., 2023). Human infections can result in severe illnesses, including hemorrhagic fever with renal syndrome (HFRS) and hantavirus pulmonary syndrome (HCPS), which can be fatal (Jiang et al., 2017; Vial et al., 2023). Hantaviruses have a worldwide distribution and are prevalent in Asia, Europe, and the Americas, causing approximately 20,000–100,000 cases annually (Avšič‐Županc et al., 2019; Jiang et al., 2017; Zhang et al., 2010). In the Americas, hantaviruses cause approximately 300 cases of HCPS annually (Vial et al., 2023). Although the seroprevalence and case rates of hantaviruses in the Americas are lower than in other regions, the case mortality and disease severity are higher (Vial et al., 2023). Andes orthohantavirus (ANDV), present in Argentina and Chile, is the only hantavirus with documented human‐to‐human transmission and has comparatively high case mortality rates (Hjelle & Torres‐Pérez, 2010; Vial et al., 2023). In Chile, there is one known reservoir rodent species for ANDV, *Oligoryzomys longicaudatus*, and six other known rodent hosts (*Abrothrix olivaceus*, *Phyllotis darwini*, *Abrothrix longipilis*, *Rattus rattus*, *Loxodontomys micropus*, and *Rattus norvegicus*) that have shown evidence of infection or exposure (Llanos‐Soto & González‐Acuña, 2019). Cross‐species transmission of hantavirus from the primary reservoir host to secondary hosts has been documented in various hantaviruses (Delfraro et al., 2008; Medina et al., 2009; Vapalahti et al., 1999; Weidmann et al., 2005) and may play a role in human disease emergence (Kreuder Johnson et al., 2015; Olival et al., 2017).

For hantaviruses to spread from one rodent species to another, there must be ecological interaction between individuals of different rodent species, which can constitute aggressions, sharing of resources, or co‐habitation (Palma et al., 2012). In Chile, the primary transmission mode for ANDV between rodent species is through saliva, but can also include urine and feces, which are shared when rodents interact (Padula et al., 2004). In the case of ANDV, uncovering the ecological factors underlying cross‐species viral transmission could be particularly informative because the mode by which hantaviruses have diversified in South America is still unclear (Kuhn & Schmaljohn, 2023; Rivera et al., 2015). The limited understanding of hantavirus evolution in South America restricts our capacities to anticipate zoonotic and cross‐species spillover transmission.

Understanding the factors driving pathogen transmission and identifying potential hosts in the hantavirus transmission cycle in wildlife is essential for mitigating the risk of zoonotic spillover (Cleaveland et al., 2007; Hjelle & Torres‐Pérez, 2010). Network analysis has emerged as a valuable tool in ecology, offering insights into the complex species interactions within ecosystems (Poulin, 2010; Proulx et al., 2005). Network analysis provides a framework for studying ecological systems by representing the relationships among different components and can be a valuable tool for studying wildlife disease ecology and pathogen transmission (Craft & Caillaud, 2011; Silk et al., 2017). Ecological networks depict communities by representing parts of the community as nodes (individuals or groups) that are connected by edges (an interaction or shared characteristic linking nodes; Craft & Caillaud, 2011). Factors contributing to pathogen transmission and spillover can be explored by reconstructing host networks (Bordes et al., 2017; Craft & Caillaud, 2011).

One challenge in constructing ecological networks is determining how nodes, in this case, potential hosts, should be connected to best represent species or individual interactions (Craft & Caillaud, 2011). Sharing of ectoparasites can serve as an informative link between two hosts in a network (Poulin, 2010) because it serves as a proxy for ecological interaction between hosts (Nieberding & Olivieri, 2007). Ectoparasites can be generalists (i.e., parasitizing many host species) or specialists (i.e., parasitizing one or very few host species) (Poulin, 2007). Host biology is an essential factor in whether an ectoparasite can successfully parasitize the host (Poulin, 2007). When hosts have an ectoparasite association in common, it indicates interaction or similarity between hosts (Poulin et al., 2011). Phylogenetic relatedness, habitat or range overlap, morphology, and phenology are some similarities that hosts may share when they share ectoparasites (MacDonald & Brisson, 2022; Poulin et al., 2011; Runghen et al., 2021; Sun et al., 2023). Ectoparasite sharing between hosts differs based on the parasite species. Nevertheless, parasite sharing can be broadly categorized into parasite species that require direct contact to be shared, such as lice, nidicolous ticks, and some mites, and species that can be shared indirectly through shared environments, such as non‐nidicolous ticks, some mites, and fleas (Di Giovanni et al., 2021). Regardless of how ectoparasites are transmitted between host species, the sharing or trait of being parasitized by two of the same ectoparasite species can indicate ecological similarity between hosts.

Based on ecological, biogeographic, or evolutionary similarities, estimating ectoparasite sharing between potential host species in a pathogen transmission network could be a powerful tool to infer host interaction. In the case of ANDV cross‐species spillover transmission, ectoparasite sharing may be particularly relevant because ANDV transmission between rodents requires direct host contact or sharing of resources which can be well represented by sharing ectoparasite species. Beyond ANDV, the approach of using a parasite‐sharing network can help translate data from one study system into another. The parasite‐sharing network is specifically useful for pathogen transmission systems, especially those that depend on ectoparasites, because there may be little data on how potential hosts interact due to the difficulty of collecting behavioral data. At the same time, there can be an abundance of other ecological data (i.e., ectoparasite associations for those hosts) that can help bridge the gap for understanding host interactions. Using host–parasite interactions to create a network structure and study interactions is not novel (Bordes et al., 2017; Dáttilo et al., 2020; Runghen et al., 2021). Nevertheless, previous approaches tend to include ectoparasites in the network in the same way that hosts are included (i.e., both parasites and hosts are included as nodes; Dáttilo et al., 2020, Runghen et al., 2021). Here, we demonstrate how known ectoparasite associations can be utilized to infer host interactions, as connections between nodes, to infer pathogen transmission. This novel transmission network provides a framework for viewing ectoparasite sharing as a host trait independent of which specific parasite species are being shared. In this framework, we lose knowledge of which ectoparasite species are contributing to interactions, but we gain the ability to more clearly visualize how hosts interact, how a host and parasite species ensemble is structured, and the strength of interactions between hosts.

In this study, we investigated the evolutionary and ecological factors that predict known and potential novel hantavirus hosts using network analysis on a dataset of rodent–ectoparasite records in Chile. To uncover interactions and possible transmission dynamics between hosts, we hypothesize that shared ectoparasites are a proxy for the ecological interactions among host species which can be used to build a host interaction network. By examining the connections and centrality of rodent species within the ectoparasite network, we aim to uncover the ecological, phylogenetic, and biogeographic drivers of host centrality and cross‐species transmission events. Ultimately, this research adds to a growing body of work showing the applications of network analysis in disease ecology which can inform targeted surveillance efforts to specific rodent species and in specific regions and inform the mitigation of hantavirus emergence.

## 2 MATERIALS AND METHODS

### 2.1 Host–parasite association dataset

We constructed an updated dataset of published rodent–ectoparasite associations in Chile. We conducted a search of rodent–ectoparasite reports in Chile published from January 2015 to March 2023 in PubMed and Web of Science. We conducted our search on March 10, 2023. We used the same search terms as the original review: ((“Acari” OR “Ixodida*” OR “Phthiraptera*” OR “Siphonaptera*” OR “tick*” OR “mite*” OR “lice” OR “louse”) AND (Chile)) AND (Rodent* OR Rodentia). Our inclusion criteria required reports to be original (i.e., not a review), conducted in Chile, and include collection of rodents and ectoparasites. We used Covidence software (www.covidence.org) to streamline the filtering, eligibility, and data extraction process. Findings were used to complement and update the Landaeta‐Aqueveque et al. (2021) dataset of host–ectoparasite associations in Chile. We corrected rodent names according to the taxonomic standing in the Integrated Taxonomic Information System (ITIS, 2023; www.itis.gov) and removed any genus‐level ectoparasite or rodent record.

### 2.2 Host geographical range

We obtained geographic range data of rodents of Chile using the International Union for Conservation of Nature (IUCN, 2023) database with the search criteria to include results with taxonomy set to “Rodentia” and land area to “Chile.” We used the administrative boundary for Chile from DIVA‐GIS (http://www.diva‐gis.org/gdata) to restrict the IUCN ranges to Chile. For the seven rodent species with documented ectoparasite associations that did not have distributional range data available in IUCN, we searched GBIF (GBIF.org, 2024) for occurrence data to build an approximate distributional range.

We directly downloaded range data from IUCN for the rodent species available. For those from GBIF (datasets: GBIF.ES, 2023; Oyaneder et al., 2023), we used the R package *gbif.range* (Chauvier et al., 2022.) to generate approximate distributional ranges. Occurrence data from GBIF were downloaded on March 6, 2024. Using spatial analysis packages *sf* (Pebesma & Bivand, 2023), *raster* (Hijmans, 2023), and *phyloraster* (Alves‐Ferreira et al., 2024) in R version 2022.12.0 (R Core Team, 2023), we constructed species richness maps for the 67 rodent species recorded in Chile and the subset of 45 rodent species with ectoparasite associations. We then calculated the geographic overlap for rodent species using Jaccard's similarity index (Jaccard, 1901) with the *sf* package in R where we used the function, “st intersection” (Pebesma & Bivand, 2023), to obtain the area of overlap between the two species. Following the equation for Jaccard's similarity, we divided the intersection by the total of both ranges minus the intersection. We also used the *sf* package in R to calculate geographic distance using range centroids between rodent species.

### 2.3 Host phylogenetic relatedness

To calculate the pairwise phylogenetic distance for the rodent species in our network, we accessed the mammalian phylogeny (Upham et al., 2019) through the VertLife project (https://vertlife.org/). We used the subset tool to request 1000 trees for only the rodent species in our rodent–ectoparasite network (50 rodents in the network and 48 available through VertLife). Using the *ape* package in R, we built the most probable tree based on the 1000 trees from for the species available in VertLife. Using *ape*, we also calculated the pairwise phylogenetic (patristic) distance, with the “cophenetic” function, for the rodents in our network for downstream analysis.

### 2.4 Ectoparasite sharing

We assessed how geographic and phylogenetic factors influence rodent connections using two methods: (1) probability as a function of geographic and genetic relatedness to share ectoparasites using logistic regressions and (2) correlation of phylogenetic and geographic distances with network path distances using Mantel tests (see Section 2.5). We used R software version 2022.12.0 (R Core Team, 2023) for all statistical analyses and considered results with *p* < 0.05 to be significant. First, we calculated the probability of rodent species to share an ectoparasite based on pairwise phylogenetic distance, geographic overlap, and geographic distance between rodent species using the *geotax* package in R version 2022.12.0 (Robles, 2023; Robles‐Fernández & Lira‐Noriega, 2017). We excluded ectoparasites associated with less than three rodent species from the probability analysis to mitigate uncertainty related to low sample size, as in Robles‐Fernández and Lira‐Noriega (2017), which used the same threshold and similar to Dáttilo et al. (2020), which excluded those with less than one association. Then, we constructed pairwise interaction matrices representing the phylogenetic distance, geographic overlap, and geographic distance between rodent species. We then constructed binary interaction matrices between ectoparasites and rodent species with “1” representing a documented association between a rodent and an ectoparasite and “0” representing no interaction. Using these interaction matrices, we calculated logistic regression coefficients drawn from a distribution of 1000 permutations with phylogenetic distance, geographic overlap, or geographic distance as the predictor variable for the sharing of ectoparasites, the binary interaction, between two rodent species. We calculated logistic regression coefficients for each ectoparasite species and for the overall species pooled for each predictor. With the logistic regression coefficients, we calculated the probability of rodent species to share ectoparasites based on each predictor. We used the *geotax* package in R version 2022.12.0 (Robles, 2023; Robles‐Fernández & Lira‐Noriega, 2017) to create the binary interaction matrices and to calculate logistic regression coefficients and probability vectors.

### 2.5 Network analysis

We built a network estimating how rodents were connected through the ectoparasites they share using Gephi software (Bastian et al., 2009). We first built a weighted interaction matrix to show the number of ectoparasites shared by each pair of rodents. With this, we built a weighted network in Gephi using *Fruchterman Reingold* followed by *Force Atlas 2* visualization algorithms with rodents as nodes and shared ectoparasites as edges (Bastian et al., 2009). In our bipartite network structure, the rodent hosts are included as nodes and the ectoparasites they share are the edges connecting them. With this network structure we lose the information of which ectoparasite species are being shared and all species are treated equally in their contribution to the relationship between two rodents. We chose this structure because we are primarily focused on the rodent hosts, using the ectoparasites as a tool by which to connect them as a proxy of likely direct or indirect interaction.

From the rodent–ectoparasite network model, we extracted the closeness centrality of each rodent species in Gephi. The closeness centrality measures the average distance from one node to all other nodes in the network and provides a concept for how central or key a species is in the community (Brandes, 2001). Closeness centrality is an appropriate metric as a proxy of interaction because we are using a diverse set of ectoparasite species as edges, which are shared directly and indirectly. Each connection may not have to be direct for rodents to share ectoparasites but could happen through the environment or shared habitat. Our pathogen of interest, ANDV, may be transmitted between rodent species through habitat or resource sharing and not necessarily only through direct contact. This supports the use of closeness centrality in both cases because closeness centrality goes beyond traditional network metrics and considers spread through a network in a mode that is not entirely reliant on immediate interactions (Bloch et al., 2023).

We also generated a path distance matrix for the network using the package *igraph* in R which calculates the shortest path between each pair of nodes (Nepusz, 2006). The path distance matrix is a pairwise matrix with rodents as column and row headers, where the interaction between columns and rows is the number of ectoparasite species (edges) needed to connect each pair of rodent hosts (nodes). Using this pairwise path matrix, we tested for correlation between path length with both phylogenetic and geographic distance using a Mantel test in the R package *vegan* (Oksanen et al., 2022). We tested correlation from the pairwise matrices of path length versus phylogenetic distance, path length vs. geographic distance, and geographic vs. phylogenetic distances.

### 2.6 Spillover potential

We tested how the closeness centrality of our network model predicted hantavirus hosts using a phylogenetic logistic regression for binary‐dependent variables with closeness centrality as a continuous predictor and hantavirus host status as the binary‐dependent variable (Ives & Garland Jr., 2010). This regression model accounts for the non‐independence of the host species and evaluates how a binary trait, in this case being a hantavirus host, changes and is correlated according to both the phylogeny and the continuous variable, closeness centrality. We implemented this model using the package *phylolm* in R with the algorithm from Ho and Ané (2014). To explain the model summary, we follow the interpretation of α from the example in Ives and Garland Jr. (2010) as well as that suggested by Cooper et al. (2016) using the phylogenetic half‐life. We also applied a quantile threshold to closeness centrality to split rodents into four groups of similar closeness centrality. Based on these groups, we predicted that the high centrality would represent likely hantavirus hosts and tested this using cumulative binomial probability. For the cumulative binomial probability test (Loader, 2000), the number of species in the highest quantile was considered the number of trials, the rodent species known to be hantavirus host were the successes, and the proportion of species in the highest quantile from the entire number of rodent species studied was considered the probability of success.

### 2.7 Hantavirus in the network

We examined the relationship between rodent‐hantavirus sharing with geographic overlap and distance, and phylogenetic distance using the same methodology as described for ectoparasite sharing. For this, we added the hantavirus associations between each rodent host to the rodent–ectoparasite dataset. From this, we obtained logistic regression coefficients and probability vectors for the relationships between hantavirus host status with phylogenetic relatedness, geographic distance, and geographic overlap. We compared how hantavirus sharing and ectoparasite sharing are related to the geographic overlap and distance and phylogenetic relatedness of their rodent hosts.

## 3 RESULTS

**FIGURE 1:**
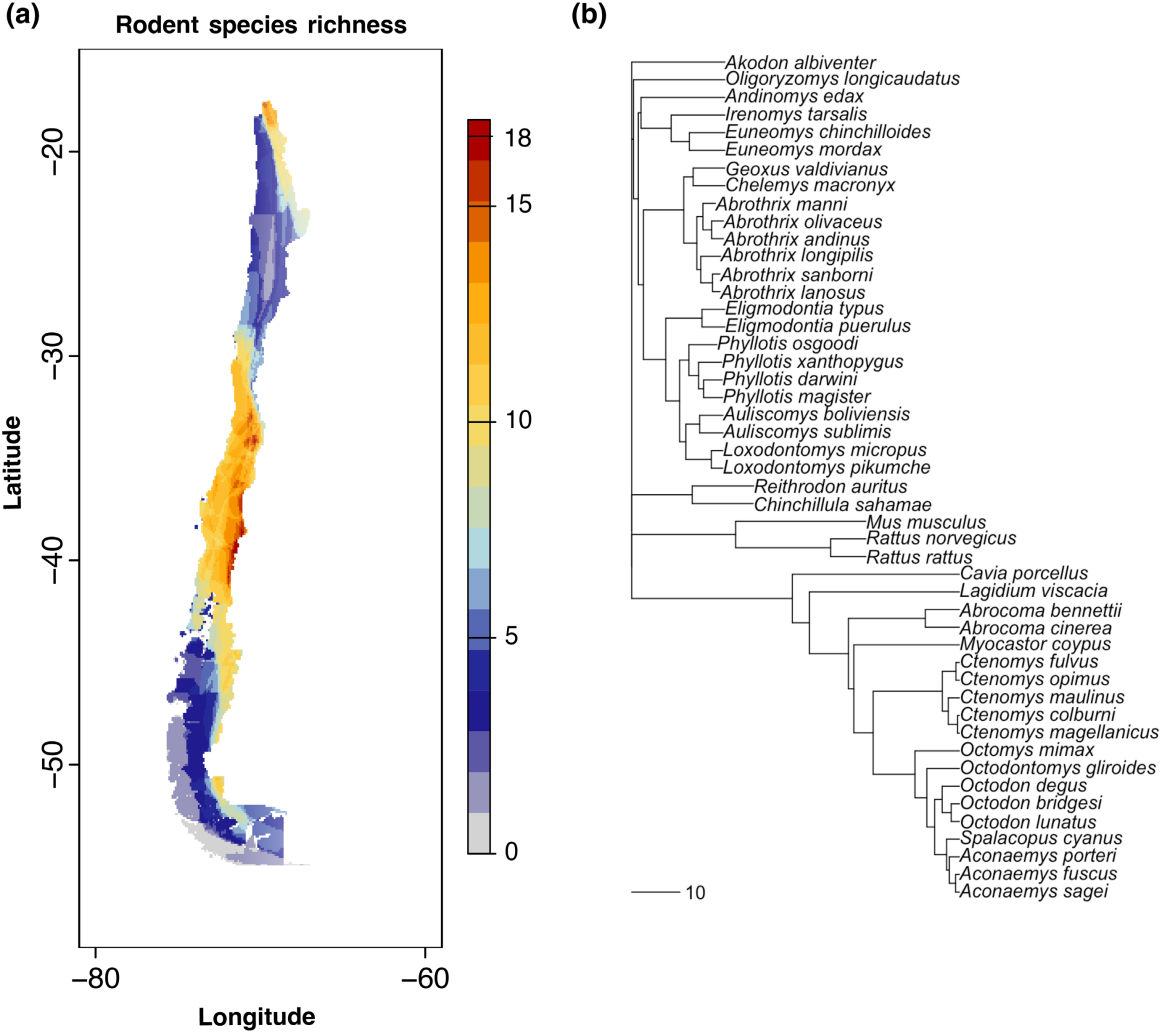
Rodent species diversity in Chile. (a) Rodent species richness for species with documented ectoparasite associations. (b) Rodent phylogeny for species with documented ectoparasite associations with maximum likelihood bootstrap percentages.

Our search resulted in six new reports of ectoparasites on rodents in Chile, with a total of 33 papers between the original review (Landaeta‐Aqueveque et al., 2021) and our update (Table S1). This created a dataset with 50 rodent species parasitized by 131 ectoparasites (91 fleas, 18 mites, 17 lice, and five tick species) and 376 unique interactions between them (Table S1). From the review, we updated seven species names according to valid ITIS names (Table S2). Of the 50 rodent species documented with ectoparasites, data were available to construct a species richness map for 45 rodents (43 from IUCN and two from GBIF; Figure 1a) and a phylogenetic tree for 48 rodents (Figure 1b). We found that the highest rodent species richness in Chile covered latitudes of 30° S to 55° S (Figure 1a; Figure S1). Our phylogenetic tree included 26 genera representing 48 species of rodents.

### 3.1 Ectoparasite sharing

**FIGURE 2:**
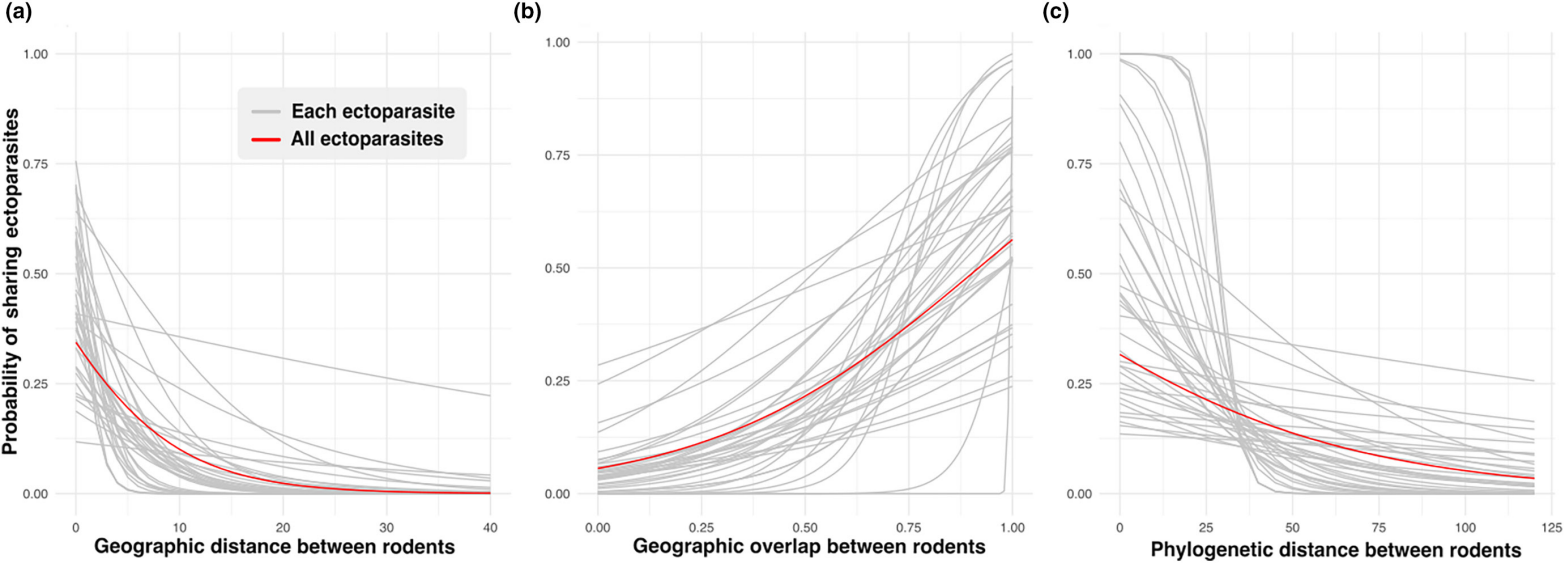
Association between sharing of ectoparasites and potential transmission drivers. Each panel shows the logistic regression curves for each ectoparasite (gray) and the overall model (red). We considered ectoparasites with three or more associations, using phylogenetic distance, geographic distance, or geographic overlap as the predictor variable. (a) Ectoparasites sharing based on phylogenetic distance between rodents. (b) Ectoparasites sharing based on geographic distance between the centroid of two rodent species. (c) Ectoparasites sharing based on the geographic overlap of rodent distributions.

We found that phylogenetic distance, geographic overlap, and geographic distance were predictors for rodents to share ectoparasites (Table 1). More closely related rodent species were overall more likely to share ectoparasite species and each individual ectoparasite also followed this trend with negative slopes for each regression (Table 1: overall models, all ectoparasites: Table S4). Sharing of ectoparasites was also strongly predicted by geographic overlap and geographic distance between rodent species (Table S4). The overall trends followed for each individual ectoparasite with positive slopes for geographic overlap and negative slopes for geographic and phylogenetic distance (Figure 2). For the phylogenetic regression, 37 species of ectoparasites and 48 species of rodents were included and for the geographic regressions 37 species of ectoparasites and 45 species of rodents were included based on available data and our limiting the analysis to ectoparasites with three or more rodent host associations. Based on the slopes of the regression model, phylogenetic distance was the strongest predictor for sharing ectoparasites, followed by geographic overlap, and then geographic distance. Individual ectoparasites followed the trends to different degrees, indicating strong or weak relationships with geographic and genetic factors.

**TABLE 1:** Logistic regression coefficients and summary for ectoparasite sharing.

	Geographic overlap	Geographic distance	Phylogenetic distance
$\beta_{0}$	−2.82881254	−0.6467294	−0.769268852
Std. Error	0.11842025	0.10890083	0.133686998
*z* value	−23.8897436	−5.9419812	−5.75342839
Pr(>|*z*|)	1.66E−124	7.21E−07	3.41E‐06
2.50%	−3.06091196	−0.8601711	−1.031290552
97.50%	−2.59671312	−0.4332877	−0.507247152
$\beta_{1}$	3.07615777	−0.1541742	−0.021322972
Std. Error	0.20441326	0.01446215	0.002463259
*z* value	15.0478327	−10.63058	−8.637090339
Pr(>|*z*|)	8.95E−44	3.77E−18	1.06E−10
2.50%	2.67551514	−0.1825195	−0.026150871
97.50%	3.47680041	−0.1258289	−0.016495074

*Note*: The overall logistic regression model coefficients for each of the predictor variables (phylogenetic relatedness, geographic distance, and geographic overlap) are reported with their summary statistics.

### 3.2 Rodent–ectoparasite network

**FIGURE 3:**
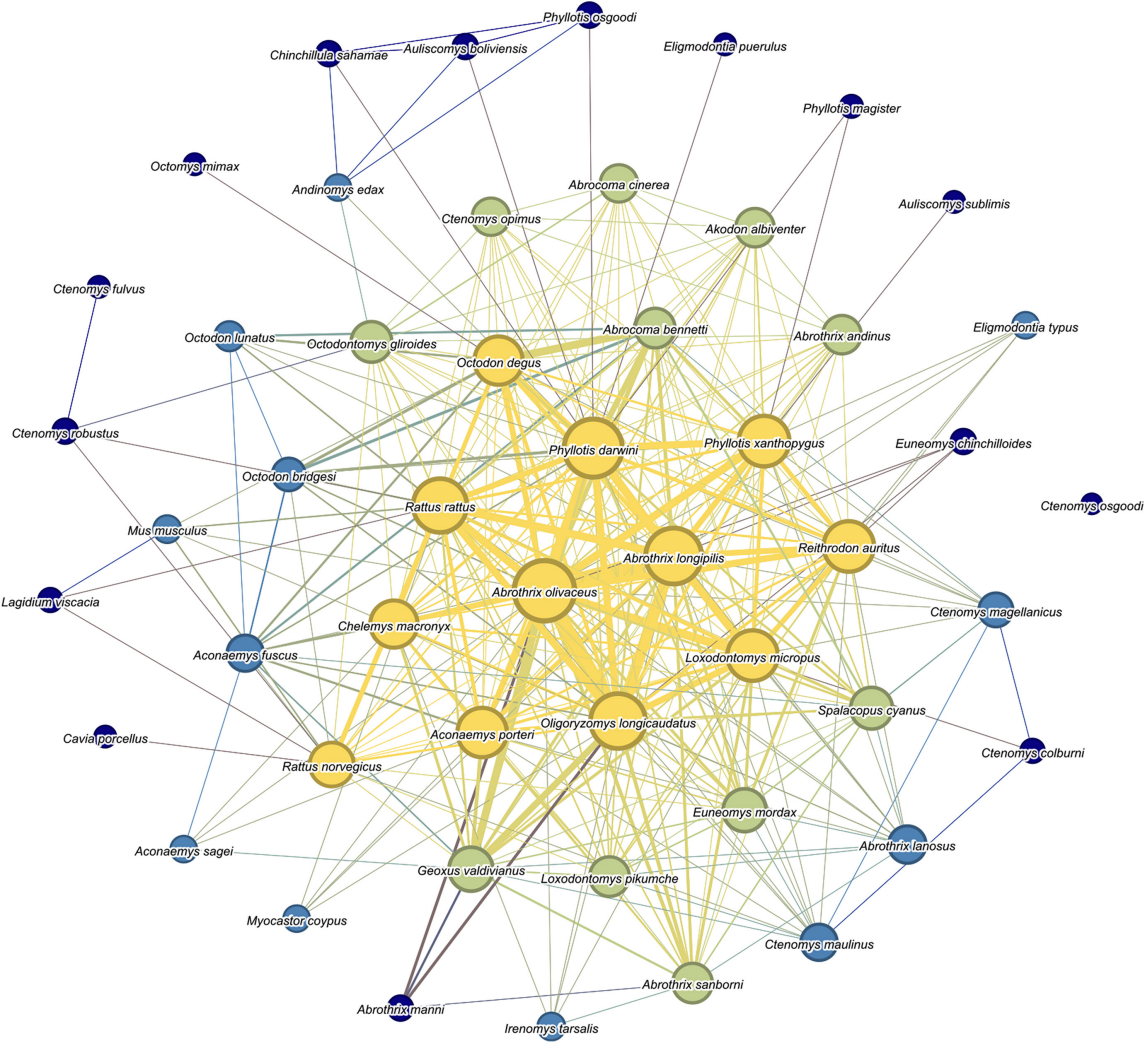
Interaction network model. The network model depicts the 376 unique interactions (edges) between 50 rodent hosts (nodes) through shared ectoparasites where interactions are weighted on the number of shared ectoparasites between rodents. Node size: Closeness centrality of each rodent Edge size: Number of shared ectoparasites between two rodent species. Yellow‐Green‐Blue‐Dark Blue: Decreasing closeness centrality quantiles based on levels of rodent‐parasite sharing.

**FIGURE 4:**
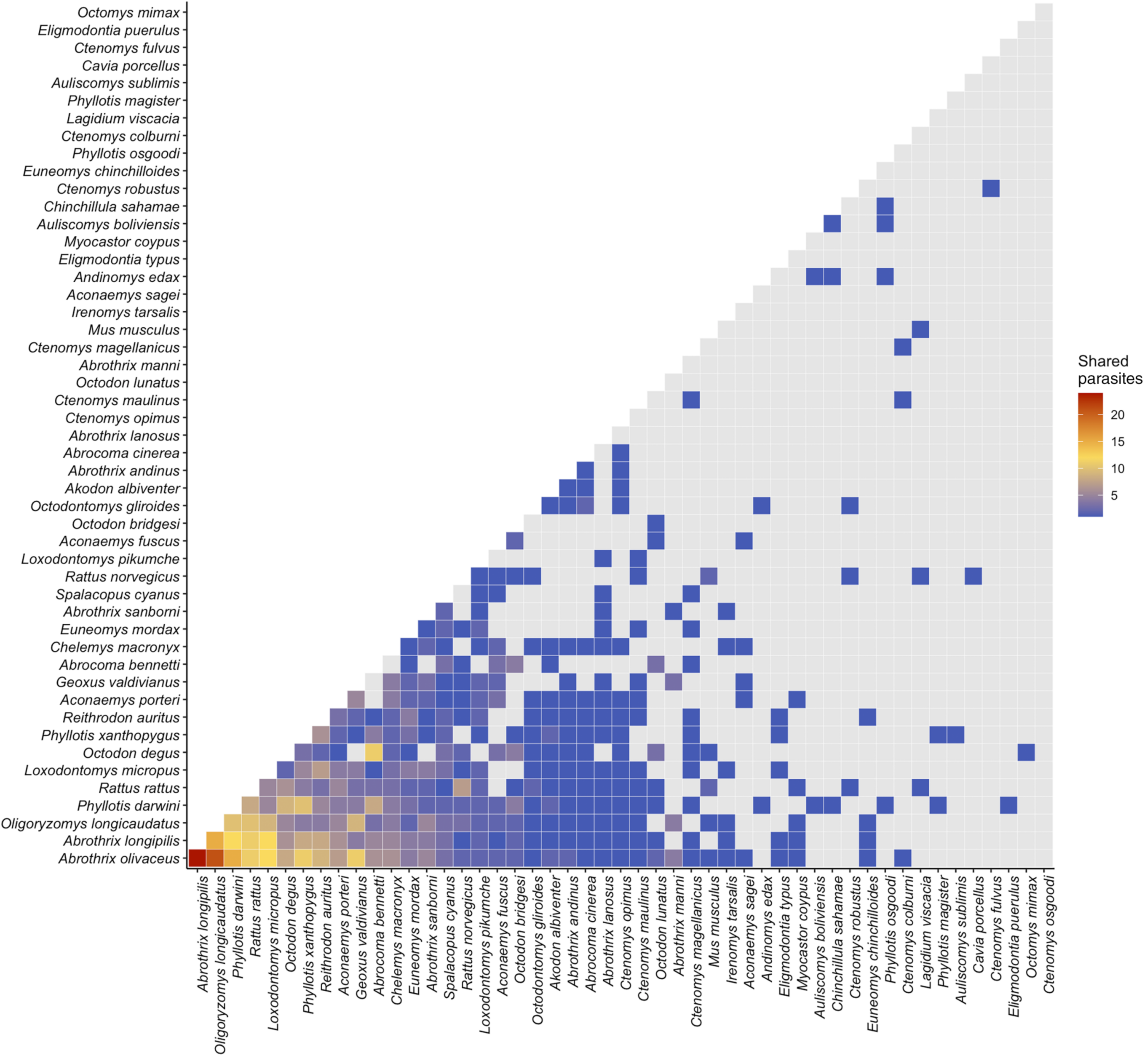
Interaction matrix among rodent species. The number of shared ectoparasites between each pair of rodents in Chile are ordered from those sharing the most ectoparasites (red) to those sharing the least (blue) or none (gray).

**FIGURE 5:**
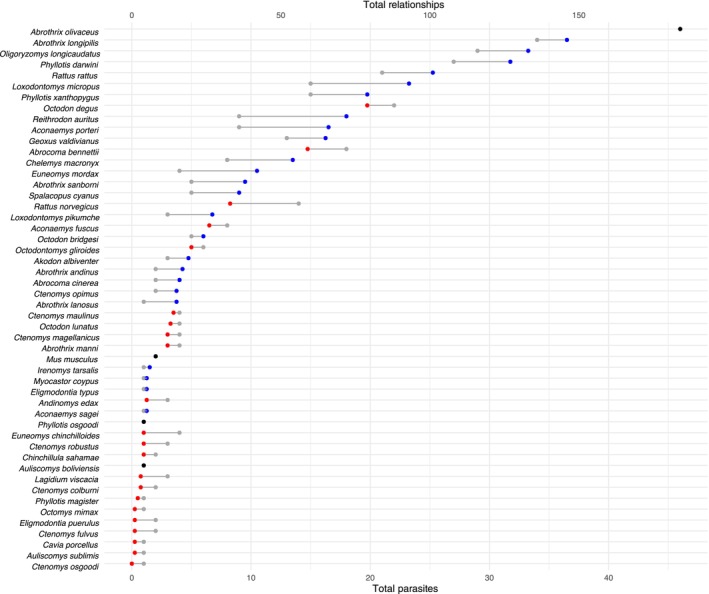
Rodent–ectoparasite association. Left axis: rodent species Bottom axis: Total number of parasite associations represented by the gray points for each rodent Top axis: Total number of connections to other rodents through ectoparasites. The gray point shows the number of ectoparasite associations each rodent has individually (bottom) and the colored point represents the total number of relationships to other rodents through shared ectoparasites (top). Blue points indicate that the rodent is connected to more than average (~4 connections per ectoparasite), red indicated less than average, and black is average.

We included all 50 rodent and 131 ectoparasite species, with 376 unique interactions between rodents through ectoparasites, in our network model (Figure 3). In the rodent–ectoparasite network, we found that individual rodent species were parasitized by up to 46 ectoparasite species with a maximum of 186 unique relationships to other rodents through ectoparasites. The strongest connection in our model was between *A. olivaceus* and *A. longipilis*, which shared 24 ectoparasite species (Figure 4). The overall dataset indicated a median ectoparasite‐to‐relationship ratio of 4.3, meaning that on average, each ectoparasite species connects its host to about four other hosts (Figure 5). Quantiles of network closeness centrality (CC) varied from CC = 0 (*Ctenomys osgoodi*, one ectoparasite, and no relationships) to CC = 0.78 (*A. olivaceus*, 46 ectoparasites, 184 relationships). Similarly, for matrix correlations, we found that path length in the rodent–ectoparasite network was correlated with both geographic distance (Mantel test, *n* = 44 rodent species, *p* = .001, *r* = .363) and phylogenetic distance (Mantel test, *n* = 47 rodent species, *p* = .025, *r* = .115). We found no association between geographic and phylogenetic distance (Mantel test, *n* = 44 rodent species *p* = .945, *r* = −.071). Although we had geographic data for 45 species and phylogenetic for 48 species, we did not include *Ctenomys osgoodi* in the mantel tests because it had no ectoparasite connections to other rodent species, making the total species included one less than that which we had available data.

### 3.3 Spillover prediction

**FIGURE 6:**
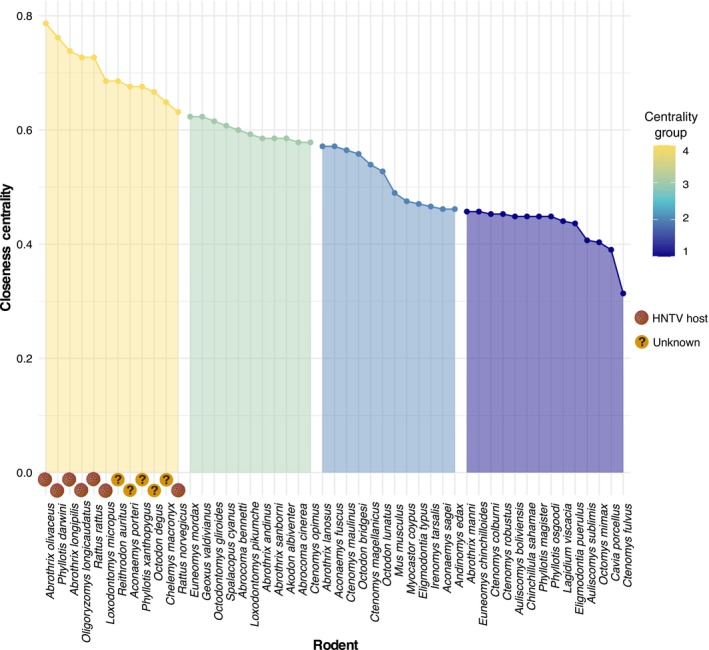
Spillover transmission risk categories based on the centrality of hosts in the rodent–ectoparasite network. Rodent species are grouped based on closeness centrality quantiles from the rodent–ectoparasite network which is used to predict spillover transmission risk. Rodent species quantile groups are shown in order of decreasing closeness centrality quantile by color: yellow, green, light blue, and dark blue. The highest quantile group in yellow includes all known hantavirus hosts (Brown dots: known hantavirus hosts) and five potential hosts (Question mark: predicted potential hantavirus hosts).

Closeness centrality was a significant predictor for rodents to be hantavirus hosts and there was very little phylogenetic signal for this trait (Closeness centrality: *p* = .002, Phylogenetic signal: α= 0.0005, a = − 3.340, $t_{\frac{1}{2}}$ = 1498, mean tip height = 40.367). Closeness centrality quantiles identified rodents prone to cross‐species hantavirus transmission, including 12 highly connected rodents (Figure 6; *A. olivaceus*, *P. darwini*, *A. longipilis*, *O. longicaudatus*, *R. rattus*, *L. micropus*, *Reithrodon auritus*, *Aconaemys porteri*, *Phyllotis xanthopygus*, *Octodon degus*, *Chelemys macronyx*, and *R. norvegicus*). Independent data from our rodent–ectoparasite network revealed that known hantavirus hosts were successfully predicted better than by chance according to a cumulative binomial probability test (*p*(*x* = 7) = 0.009). Mapping the predicted hantavirus hosts revealed hotspots of spillover transmission risk found in Chile between latitudes 45° S and 55° S in Chile (Figure S1A).

Considering known relationships between rodents and hantaviruses revealed that this interaction follows the same trends as the rodent–ectoparasite interactions. That is, the probability for a pair of rodent species to share hantavirus increased with geographic overlap and decreased with geographic and phylogenetic distance. Hantavirus sharing had stronger relationships to all three predictor variables than overall ectoparasite sharing (Table S4).

## 4 DISCUSSION

Host networks have been used in wildlife disease ecology to understand pathogen transmission, but often rely on social interactions between hosts, which can be difficult to define or collect in many systems (Craft & Caillaud, 2011). We posit that ectoparasites can act as a proxy for ecological interaction between hosts, which has been proposed previously (Nieberding & Olivieri, 2007) and is based on literature that ectoparasites are indicative of host phenology (MacDonald & Brisson, 2022), phylogenetics and geographic distribution (Poulin et al., 2011), morphology (Sun et al., 2023), and other traits (Poulin, 2007; Runghen et al., 2021). We built a host network using this concept of ecological interaction through ectoparasites to understand how other parasites or pathogens may move through a host species assemblage. Using a well‐understood rodent system in the Neotropics, we identified underlying predictors for rodents to share ectoparasites, built a weighted interaction network, and used network centrality to predict hantavirus hosts. We successfully predicted all known hosts of hantavirus in Chile and identified five rodent species as potential hantavirus reservoirs. Proximity in geographic range and phylogenetic relatedness are strong predictors for rodents to share ectoparasites and to be more connected in the network. Our findings revealed hotspot areas to inform surveillance of hantavirus in rodents in Chile.

A pair of rodent species were more likely to be parasitized by the same ectoparasite if they were closely phylogenetically related, overlapped more in range, and had a shorter distance between the center of their ranges. The trends between geographic and genetic factors with parasite sharing in the rodent–ectoparasite system are similar to trends in other systems, including viruses in bats (Wang et al., 2023), helminth and microparasites in rodents (Bordes et al., 2017), *Bartonella* in bats (McKee et al., 2019), avian malaria in bird communities (Clark & Clegg, 2017), plants and beetle species (Robles‐Fernández & Lira‐Noriega, 2017), and small mammals and ectoparasites (Dáttilo et al., 2020; Martinů et al., 2018). We expand on the understanding of how evolutionary and ecological factors influence host interactions by exploring their role in a network of hosts versus in individual host relationships. Based on our regression analysis, we found that ectoparasite sharing between individual rodents had a stronger relationship with geographic distance and overlap than with phylogenetic distance. In agreement, the network path distances were also shorter for rodents that were more geographically proximal than those that were more closely phylogenetically related. These findings contribute to both general frameworks for understanding ectoparasite–host communities and parasite sharing, and specifically to the hantavirus system in Chile.

From the rodent–ectoparasite network, we found that closeness centrality can infer hantavirus host status. Closeness centrality showed a significant correlation with hantavirus host status when there was very little phylogenetic signal, implying that using the ectoparasite‐network model is a significantly better tool than phylogenetic relationships alone for predicting hantavirus host status. We found that the 12 rodent species grouped in the quantile of high closeness centrality included all seven known hantavirus hosts and five potential hosts. It has been suggested previously that highly connected rodents in a network may play a role in zoonotic disease spillover (Bordes et al., 2017). We support high network centrality as a driver of spillover in the rodent‐hantavirus system. Using the 12 most central rodents, we identified the south‐central region of Chile as having the greatest richness of known and potential hantavirus host and reservoir species. This area has previously been defined as a mammalian biodiversity hotspot (Hernández‐Mazariegos et al., 2022), which could place it at a higher risk for emerging zoonotic diseases (Allen et al., 2017). The rodent species and areas identified can be used to inform surveillance programs aiming to identify novel hosts and regions where the virus could emerge. Identifying novel hosts and high‐risk areas for hantavirus spillover in Chile is critical as the ANDV hantavirus circulating in Chile has revealed a risk of human‐to‐human transmission (Martinez‐Valdebenito et al., 2014) and is also associated with the second highest case mortality rate for all hantaviruses worldwide (Vial et al., 2023). Anticipating and assessing for cross‐species transmission events of hantavirus in Chile can allow assessment of ANDV evolution in transmissivity and virulence, which is fundamental for early detection of enhanced pathogenicity and pandemic risk.

Pathogens and parasites can be specialists, exploiting one or few hosts, or generalists, exploiting many hosts. Phylogenetic relatedness and geographic distance can influence the ability of a pathogen or parasite to exploit multiple hosts (Poulin et al., 2011). Within the generalist–specialist framework, some pathogens and parasites act as generalists regarding one factor while they are specialists with regard to another. This means that a parasite or pathogen can be a generalist in terms of host range but a specialist in terms of restricted geographic range, which makes classifying a parasite or pathogen into only one role reductive of its multiple interactions (Poulin et al., 2011). For emerging diseases, the definition of generalist and specialist are relevant because an understanding of parasite or pathogen specificity is essential in identifying where or in what species a parasite or pathogen has the potential to expand. For example, in our rodent–ectoparasite network, the flea *Plocopsylla crypta*, had a strong relationship with geographic proximity of hosts, but a weak relationship with phylogenetic relatedness. This indicates that *P. crypta* is more of specialist in terms of parasitizing hosts in a restricted geographic area, but within that area it can act as a generalist by parasitizing a wide range of distantly related host species. Although the majority of ectoparasites in our analysis were fleas, the next largest group, mites, notably had stronger relationships with phylogenetic relatedness and geographic proximity than fleas in general did. This may be because it is more beneficial for directly transmitted parasites, like fleas, to parasitize many different host species while it can be detrimental for indirectly transmitted parasites to do the same (Poulin et al., 2007). Using this framework can help expand the definition of generalist and specialist parasites because it considers multiple factors that influence the ability of parasites to exploit hosts.

Our findings may also be informative in deciphering how hantaviruses have diversified in South America. There are over 28 hantaviruses that can cause human disease and others of unknown zoonotic risk found in a wide range of wildlife species, including rodents, bats, moles, shrews, reptiles, and fish (Avšič‐Županc et al., 2019; Vial et al., 2023). Hantaviruses were previously thought to have co‐evolved with their hosts. Nevertheless, recent discoveries on new hosts, new viral strains, and the evolution rate of hantaviruses do not entirely support this idea (Kuhn & Schmaljohn, 2023). The possibility of preferential host switching has been proposed but is not supported in hantaviruses in South America, for which geographic proximity is a potential explanation of hantavirus diversification (Rivera et al., 2015). Using the known hantavirus hosts in Chile, we found that geographic proximity, phylogenetic relatedness, and centrality in the rodent–ectoparasite network were predictors for rodents being known hantavirus hosts. Hantavirus host status was more dependent on geographic proximity and phylogenetic relatedness than the average for ectoparasites, indicating that it is more of a specialist than most ectoparasite species. We found that hantavirus had a stronger relationship with geographic overlap and geographic distance between rodents than with phylogenetic distance. The relationships between geography and phylogeny to explain infection provide support for exploring the hypothesis that the geographic proximity of hosts has influenced the diversification of hantaviruses in South America. Host sympatry may suggest a host interaction leading to cross‐species spillover and viral diversification. Of the five species predicted to be unknown ANDV hosts, two are non‐Myomorph species (*O. degus* and *A. porteri*). Both species are in close geographic proximity and share considerable numbers of ectoparasites with known ANDV hosts, suggesting the potential for direct interaction necessary for ANDV transmission between rodents. If these species are found infected or seropositive to ANDV, such findings would validate our predictions and would represent spillover into phylogenetically distinct hosts, providing significant context to how hantaviruses have diversified in South America.

The interpretation of our results must be considered in the context of data availability and geographic extent. Rodent–ectoparasite associations were based on documented associations and may be biased toward rodent species of known health concern and not fully represent all ectoparasite associations in Chile. Geographic range and genetic data did not fully represent the 50‐rodent dataset, with five species missing geographic data and two species missing genetic data. Notably, IUCN failed to include *R. rattus* or *R. norvegicus* distributions in Chile. Nevertheless, both species have documented hantavirus infections in Chile (Lobos et al., 2005). The rodent species names and taxonomic standing that we included are according to the ITIS and may not represent recent advances in Chilean rodent taxonomy (D'Elía et al., 2020). A comprehensive dataset geographic ranges would improve our understanding of hantavirus transmission dynamics. Additionally, we limited our study area to the geographic extent of Chile. We chose this limited extent to facilitate clear boundaries for our literature review and to account for differences in sampling efforts country‐country in South America. Also, although many administrative country boundaries may not be ecologically relevant, the boundaries of Chile correspond to biogeographic regions due to the Andean cordillera on the east, the Pacific Ocean on the west, ice fields in the south, and the Atacama desert in the north (Morrone, 2018). Hantaviruses, however, are distributed globally and our approach may not scale to a global extent or in every ecosystem where hantaviruses are present.

We demonstrated that ecological networks based on shared ectoparasites can elucidate how wildlife host species interact in the transmission of parasites and pathogens. Host interactions can be challenging to estimate due to logistical or biological constraints. Our findings reveal that cross‐species transmission dynamics are influenced by host phylogeny and geographic range, which together culminate in ectoparasite sharing. To validate the predictive power of our approach we suggest expanding the species tested for hantavirus in Chile, and even targeting capture and sampling of potential hosts identified in our study. Similarly, future research should account for environmental filtering as a potential confounding factor to determine the extent to which parasite and pathogen‐spillover transmission across species and geographies is influenced by climate. Although shared ectoparasites are not a direct measure of interaction, they offer a less laborious method for connecting hosts while still being ecologically relevant. Further studies focused on multiple pathogens or parasites could use similar methods and include network communities to understand how more specialized pathogens or parasites cluster within specific hosts. Ectoparasite associations are indicative of host biology and using known interactions can help disease ecologists predict spillover transmission.

## AUTHOR CONTRIBUTIONS

**Reilly N. Brennan:** Conceptualization (supporting); data curation (equal); formal analysis (lead); investigation (lead); methodology (lead); visualization (lead); writing – original draft (lead); writing – review and editing (equal). **Sally L. Paulson:** Investigation (supporting); writing – review and editing (equal). **Luis E. Escobar:** Conceptualization (lead); data curation (supporting); formal analysis (supporting); funding acquisition (lead); investigation (supporting); methodology (supporting); supervision (lead); writing – review and editing (equal).

## CONFLICT OF INTEREST STATEMENT

We declare no competing interests.

### OPEN RESEARCH BADGES

This article has earned Open Data and Open Materials badges. Data and materials are available at https://github.com/reillybren/ChileRodents.

## Supporting information


Data S1

## Data Availability

Data and code are available from the Github repository https://github.com/reillybren/ChileRodents and in the Dryad repository: https://doi.org/10.5061/dryad.mpg4f4r7q. Supporting Information will be found in the online version of the article at the publisher's website.
