# Supplementary material for: Estimating pathogen‐spillover risk using host–ectoparasite interactions
Source: Ecol Evol. 2024 Jun 18;14(6):e11509. doi: 10.1002/ece3.11509 (PMC11184285; doi:10.1002/ece3.11509)
Supplement: Supplementary file 1 — Data S1 [file ECE3-14-e11509-s001.pdf]

Supplemental Figures and Tables

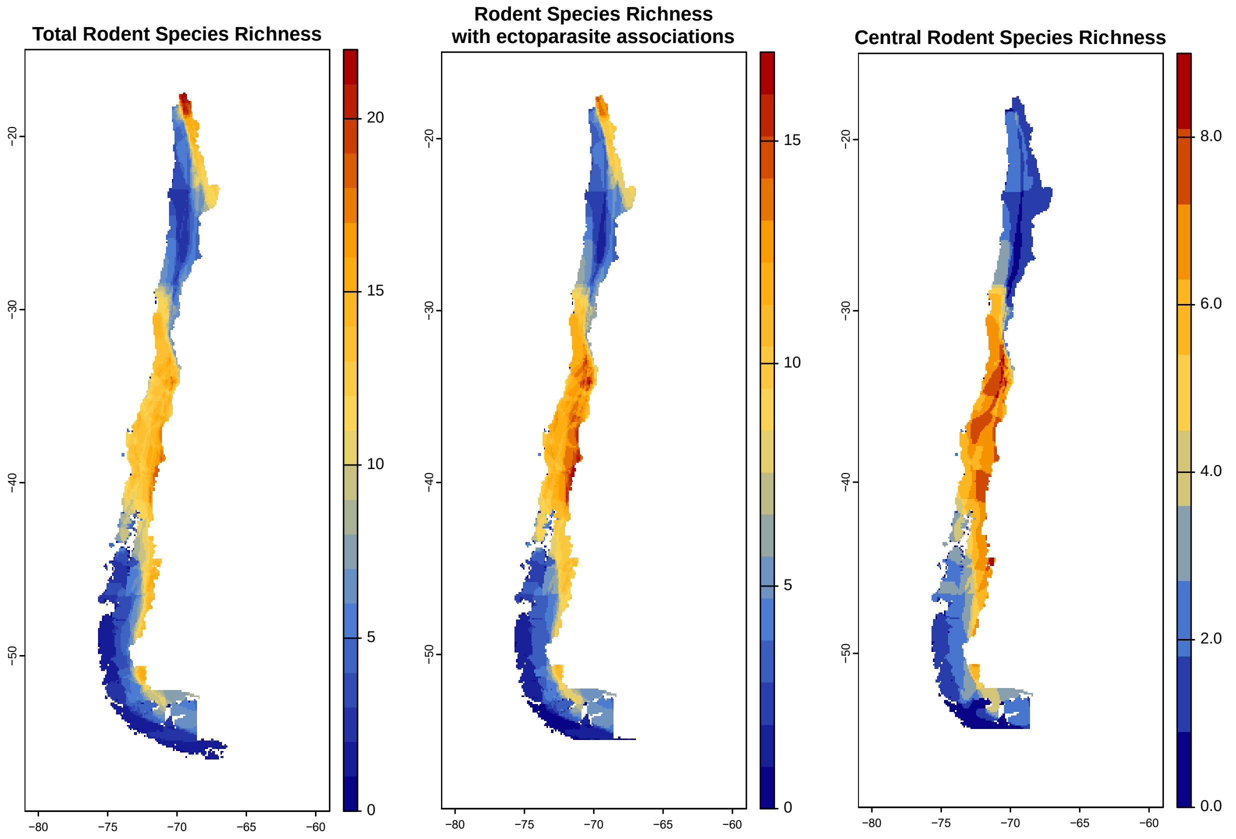

Figure S1. Rodent species richness maps of Chile: Left: All 65 rodents with range data. **Center:** The 45 rodents with ectoparasite associations and range data. **Right:** The 12 most central rodents with range data available

Table S1. Individual rodent ectoparasite associations: All individual rodent-ectoparasite associations with their references from the previous and updated review of Chilean rodents and parasites.

**Parasite**	**Rodent**	**Reference**
Abrocomaphthirus chilensis	*Abrocoma bennetti*	Durden, 2000; Gomez, 1981
Abrocomophaga chilensis	*Abrocoma bennetti*	Emerson, 1976; Yáñez-Meza, 2018
Abrocomophaga hellenthali	*Octodon degus*	Price, 2000
Agastopsylla boxi	*Abrothrix longipilis*	Beaucournu, 2014
Agastopsylla boxi	*Abrothrix olivaceus*	Beaucournu, 2014; Moreno Salas, 2020
Agastopsylla boxi	*Euneomys mordax*	Beaucournu, 2014
Agastopsylla boxi	*Loxodontomys micropus*	Beaucournu, 2014
Agastopsylla boxi	*Phyllotis xanthopygus*	Beaucournu, 2014
Agastopsylla boxi	*Reithrodon auritus*	Beaucournu, 2014
Agastopsylla guzmani	*Akodon albiventer*	Beaucournu, 2014
Agastopsylla hirsutior	*Abrothrix longipilis*	Beaucournu, 2014

Agastopsylla nylota	*Euneomys chinchilloides*	Beaucournu, 2014
Agastopsylla pearsoni	*Abrothrix longipilis*	Beaucournu, 2014
Amlistrophorus geoxus	*Geoxus valdivianus*	Sikora 2012
Androlaelaps farenholzi	*Phyllotis darwini*	Silva de la Fuente, 2020
Androlaelaps farenholzi	*Abrocoma bennetti*	Beaucournu, 2014; Veloso-Frias, 2019; Yáñez-Meza, 2018
Androlaelaps farenholzi	*Abrothrix olivaceus*	Beaucournu, 2014; Veloso-Frias, 2019; Yáñez-Meza, 2018
Androlaelaps farenholzi	*Akodon albiventer*	Beaucournu, 2014; Veloso-Frias, 2019; Yáñez-Meza, 2018
Androlaelaps farenholzi	*Phyllotis xanthopygus*	Beaucournu, 2014; Veloso-Frias, 2019; Yáñez-Meza, 2018
Argentinacarus expansus	*Abrothrix manni*	Silva de la Fuente, 2023
Argentinacarus expansus	*Abrothrix olivaceus*	Silva de la Fuente, 2023
Argentinacarus expansus	*Geoxus valdivianus*	Silva de la Fuente, 2023
Argentinacarus expansus	*Oligoryzomys longicaudatus*	Silva de la Fuente, 2023
Barreropsylla excelsa	*Abrothrix longipilis*	Beaucournu, 2014
Barreropsylla excelsa	*Abrothrix olivaceus*	Beaucournu, 2014
Barreropsylla excelsa	*Geoxus valdivianus*	Beaucournu, 2014
Barreropsylla excelsa	*Loxodontomys micropus*	Beaucournu, 2014
Barreropsylla excelsa	*Oligoryzomys longicaudatus*	Beaucournu, 2014
Chilacarus martini	*Abrothrix longipilis*	Webb, 1986
Chiliopsylla allophyla	*Abrothrix olivaceus*	Beaucournu, 2014
Chiliopsylla allophyla	*Abrothrix longipilis*	Beaucournu, 2014; Moreno Salas, 2020
Chiliopsylla allophyla	*Abrothrix sanborni*	Beaucournu, 2014
Chiliopsylla allophyla	*Chelemys macronyx*	Beaucournu, 2014
Chiliopsylla allophyla	*Irenomys tarsalis*	Beaucournu, 2014
Chiliopsylla allophyla	*Loxodontomys micropus*	Beaucournu, 2014
Chiliopsylla allophyla	*Oligoryzomys longicaudatus*	Beaucournu, 2014
Cleopsylla townsendi	*Octodon lunatus*	Beaucournu, 2014
Craneopsylla minerva	*Abrothrix olivaceus*	Beaucournu, 2014
Craneopsylla minerva	*Phyllotis darwini*	Beaucournu, 2014
Craneopsylla minerva	*Phyllotis xanthopygus*	Beaucournu, 2014
Craneopsylla minerva	*Reithrodon auritus*	Beaucournu, 2014
Ctenocephalides canis	*Rattus norvegicus*	Ruiz del Rio, 1939
Ctenocephalides felis	*Rattus norvegicus*	Beaucournu, 2014; Ruiz del Rio, 1939
Ctenocephalides felis	*Rattus rattus*	Beaucournu, 2014; Ruiz del Rio, 1939
Ctenoparia inopinata	*Abrothrix longipilis*	Beaucournu, 2014; Moreno Salas, 2020
Ctenoparia inopinata	*Abrothrix olivaceus*	Beaucournu, 2014; Moreno Salas, 2020
Ctenoparia inopinata	*Abrothrix sanborni*	Beaucournu, 2014
Ctenoparia inopinata	*Aconaemys porteri*	Beaucournu, 2014
Ctenoparia inopinata	*Geoxus valdivianus*	Beaucournu, 2014
Ctenoparia inopinata	*Loxodontomys micropus*	Beaucournu, 2014

Ctenoparia inopinata	*Oligoryzomys longicaudatus*	Beaucournu, 2014; Moreno Salas, 2020
Ctenoparia inopinata	*Phyllotis darwini*	Beaucournu, 2014
Ctenoparia inopinata	*Rattus rattus*	Beaucournu, 2014; Moreno Salas, 2019; Moreno Salas, 2020
Ctenoparia intermedia	*Loxodontomys micropus*	Beaucournu, 2014
Ctenoparia jordani	*Abrothrix longipilis*	Beaucournu, 2014; Moreno Salas, 2019; Moreno Salas, 2020
Ctenoparia jordani	*Abrothrix olivaceus*	Beaucournu, 2014; Moreno Salas, 2019; Moreno Salas, 2020
Ctenoparia jordani	*Loxodontomys micropus*	Beaucournu, 2014; Moreno Salas, 2019; Moreno Salas, 2020
Ctenoparia jordani	*Oligoryzomys longicaudatus*	Beaucournu, 2014; Moreno Salas, 2019; Moreno Salas, 2020
Ctenoparia jordani	*Rattus rattus*	Beaucournu, 2014; Moreno Salas, 2019; Moreno Salas, 2020
Ctenoparia topallii	*Abrothrix sanborni*	Beaucournu, 2014
Ctenoparia topallii	*Loxodontomys micropus*	Beaucournu, 2014
Ctenoparia topallii	*Spalacopus cyanus*	Beaucournu, 2014
Ctenoparia topallii	*Abrothrix longipilis*	Beaucournu, 2014; Moreno Salas, 2020
Ctenoparia topallii	*Abrothrix olivaceus*	Beaucournu, 2014; Moreno Salas, 2020
Ctenoparia topallii	*Oligoryzomys longicaudatus*	Beaucournu, 2014; Moreno Salas, 2020
Dasypsyllus aedon	*Abrothrix longipilis*	Beaucournu, 2014
Dasypsyllus araucanus	*Abrothrix olivaceus*	Beaucournu, 2014
Delostichus coxalis	*Abrocoma bennetti*	Beaucournu, 2014; Moreno Salas, 2020
Delostichus coxalis	*Abrothrix olivaceus*	Beaucournu, 2014
Delostichus coxalis	*Octodon degus*	Beaucournu, 2014; Moreno Salas, 2020
Delostichus coxalis	*Octodon lunatus*	Beaucournu, 2014
Delostichus coxalis	*Rattus rattus*	Beaucournu, 2014; Moreno Salas, 2019; Moreno Salas, 2020
Delostichus degus	*Abrocoma bennetti*	Beaucournu, 2014
Delostichus degus	*Octodon degus*	Beaucournu, 2014; Moreno Salas, 2020
Delostichus octomyos	*Octodon degus*	Beaucournu, 2014
Delostichus octomyos	*Octomys mimax*	Beaucournu, 2014
Delostichus phyllotis	*Abrocoma bennetti*	Beaucournu, 2014; Moreno Salas, 2020
Delostichus phyllotis	*Octodon degus*	Beaucournu, 2014; Moreno Salas, 2020
Delostichus phyllotis	*Phyllotis darwini*	Beaucournu, 2014; Moreno Salas, 2020
Delostichus phyllotis	*Octodon bridgesi*	Beaucournu, 2014; Moreno Salas, 2020

Delostichus smiti	*Abrocoma bennetti*	Beaucournu, 2014; Moreno Salas, 2020; Yáñez-Meza, 2018
Delostichus smiti	*Abrothrix longipilis*	Beaucournu, 2014; Yáñez-Meza, 2018
Delostichus smiti	*Octodon degus*	Beaucournu, 2014; Moreno Salas, 2020; Yáñez-Meza, 2018
Delostichus smiti	*Phyllotis darwini*	Beaucournu, 2014; Moreno Salas, 2020; Yáñez-Meza, 2018
Delostichus smiti	*Rattus rattus*	Beaucournu, 2014; Moreno Salas, 2019; Moreno Salas, 2020; Yáñez-Meza, 2018
Dewacarus lemuensis	*Loxodontomys pikumche*	Silva de la Fuente, 2016
Echidnophaga gallinacea	*Cavia porcellus*	Beaucournu, 2014
Echidnophaga gallinacea	*Rattus norvegicus*	Beaucournu, 2014; Yáñez-Meza, 2018
Ectinorus angularis	*Abrothrix olivaceus*	Beaucournu, 2014
Ectinorus chilensis	*Abrocoma bennetti*	Beaucournu, 2014; Moreno Salas, 2020; Yáñez-Meza, 2018
Ectinorus chilensis	*Aconaemys fuscus*	Beaucournu, 2014; Moreno Salas, 2020
Ectinorus chilensis	*Octodon degus*	Beaucournu, 2014; Moreno Salas, 2020
Ectinorus chilensis	*Oligoryzomys longicaudatus*	Beaucournu, 2014; Moreno Salas, 2020
Ectinorus chilensis	*Spalacopus cyanus*	Beaucournu, 2014; Moreno Salas, 2020
Ectinorus cocyti	*Abrocoma bennetti*	Beaucournu, 2014; Moreno Salas, 2020
Ectinorus cocyti	*Abrothrix olivaceus*	Beaucournu, 2014; Moreno Salas, 2020
Ectinorus cocyti	*Octodon degus*	Beaucournu, 2014; Moreno Salas, 2020
Ectinorus cocyti	*Spalacopus cyanus*	Beaucournu, 2014; Moreno Salas, 2020
Ectinorus curvatus	*Phyllotis darwini*	Beaucournu, 2014
Ectinorus deplexus	*Euneomys chinchilloides*	Beaucournu, 2014
Ectinorus gallardoi	*Aconaemys fuscus*	Beaucournu, 2014
Ectinorus hertigi	*Ctenomys fulvus*	Beaucournu, 2014
Ectinorus ineptus	*Eligmodontia puerulus*	Beaucournu, 2014
Ectinorus ineptus	*Phyllotis darwini*	Beaucournu, 2014
Ectinorus insignis	*Eligmodontia puerulus*	Beaucournu, 2014
Ectinorus ixanus	*Loxodontomys micropus*	Beaucournu, 2014
Ectinorus lagidium	*Lagidium viscacia*	Beaucournu, 2014
Ectinorus levipes	*Abrothrix longipilis*	Beaucournu, 2014
Ectinorus martini	*Abrothrix olivaceus*	Beaucournu, 2014
Ectinorus martini	*Aconaemys fuscus*	Beaucournu, 2014
Ectinorus martini	*Aconaemys porteri*	Beaucournu, 2014
Ectinorus martini	*Aconaemys sagei*	Beaucournu, 2014

Ectinorus martini	*Chelemys macronyx*	Beaucournu, 2014
Ectinorus martini	*Geoxus valdivianus*	Beaucournu, 2014
Ectinorus mondacai	*Aconaemys fuscus*	Beaucournu, 2014
Ectinorus mondacai	*Aconaemys porteri*	Beaucournu, 2014
Ectinorus nomisis	*Andinomys edax*	Beaucournu, 2014
Ectinorus nomisis	*Octodontomys gliroides*	Beaucournu, 2014
Ectinorus onychius	*Abrothrix longipilis*	Beaucournu, 2014
Ectinorus onychius	*Abrothrix olivaceus*	Beaucournu, 2014
Ectinorus onychius	*Eligmodontia typus*	Beaucournu, 2014
Ectinorus onychius	*Loxodontomys micropus*	Beaucournu, 2014
Ectinorus onychius	*Phyllotis xanthopygus*	Beaucournu, 2014
Ectinorus onychius	*Reithrodon auritus*	Beaucournu, 2014
Ectinorus setosicornis	*Lagidium viscacia*	Beaucournu, 2014
Ectinorus simonsi	*Octodontomys gliroides*	Beaucournu, 2014
Ectinorus splendidus	*Euneomys chinchilloides*	Beaucournu, 2014
Ectinorus uncinatus	*Andinomys edax*	Beaucournu, 2014
Ferrisella chilensis	*Octodon degus*	Moreno Salas, 2005
Ferrisella disgrega	*Octodontomys gliroides*	Moreno Salas, 2005
Gigantolaelaps wolffsohni	*Oligoryzomys longicaudatus*	Fuenzalida-Araya, 2020; Lareschi, 2010
Gigantolaelaps wolffsohni	*Rattus rattus*	Fuenzalida-Araya, 2020
Gyropus distinctus	*Abrocoma bennetti*	Yáñez-Meza, 2018; Moreno Salas, 2005; Castro, 2002
Gyropus distinctus	*Octodon degus*	Yáñez-Meza, 2018; Moreno Salas, 2005; Castro, 2002
Gyropus distinctus	*Octodon lunatus*	Yáñez-Meza, 2018; Moreno Salas, 2005; Castro, 2002
Gyropus elongatus	*Aconaemys fuscus*	Moreno Salas, 2005
Gyropus latipllicaris	*Ctenomys osgoodi*	Ewing, 1924
Gyropus parvus	*Ctenomys maulinus*	Moreno Salas, 2005
Hectopsylla cypha	*Abrothrix longipilis*	Beaucournu, 2014
Hectopsylla cypha	*Abrothrix olivaceus*	Beaucournu, 2014
Hectopsylla cypha	*Oligoryzomys longicaudatus*	Beaucournu, 2014
Hectopsylla cypha	*Phyllotis darwini*	Beaucournu, 2014
Hectopsylla cypha	*Phyllotis xanthopygus*	Beaucournu, 2014
Hectopsylla gemina	*Abrocoma bennetti*	Beaucournu, 2014; Yáñez-Meza, 2018
Hectopsylla gemina	*Phyllotis darwini*	Beaucournu, 2014
Hectopsylla suarezi	*Ctenomys robustus*	Beaucournu, 2014
Hectopsylla suarezi	*Octodontomys gliroides*	Beaucournu, 2014
Hectopsylla suarezi	*Rattus norvegicus*	Beaucournu, 2014
Hectopsylla suarezi	*Rattus rattus*	Beaucournu, 2014
Herpetacarus antarctica	*Loxodontomys micropus*	Silva de la Fuente, 2023
Herpetacarus antarctica	*Oligoryzomys longicaudatus*	Silva de la Fuente, 2023
Herpetacarus eloisae	*Abrothrix manni*	Silva de la Fuente, 2023
Herpetacarus eloisae	*Abrothrix olivaceus*	Silva de la Fuente, 2023; Silva de la Fuente, 2021
Herpetacarus eloisae	*Abrothrix sanborni*	Silva de la Fuente, 2021

Herpetacarus eloisae	*Geoxus valdivianus*	Silva de la Fuente, 2023; Silva de la Fuente, 2021
Herpetacarus eloisae	*Oligoryzomys longicaudatus*	Silva de la Fuente, 2023
Hoplopleura aitkeni	*Phyllotis darwini*	González-Acuña, 2003
Hoplopleura aitkeni	*Phyllotis xanthopygus*	González-Acuña, 2003
Hoplopleura andina	*Abrothrix andinus*	Gomez, 1981; González-Acuña, 2003; González-Acuña, 2005; Castro, 1981
Hoplopleura andina	*Abrothrix olivaceus*	Gomez, 1981; González-Acuña, 2003; González-Acuña, 2005; Castro, 1981
Hoplopleura andina	*Geoxus valdivianus*	Gomez, 1981; González-Acuña, 2003; González-Acuña, 2005; Castro, 1981
Hoplopleura pacifica	*Rattus norvegicus*	González-Acuña, 2003
Hoplopleura travassosi	*Oligoryzomys longicaudatus*	González-Acuña, 2003; González-Acuña, 2005
Ixodes abrocomae	*Abrocoma bennetti*	Guglielmone, 2010
Ixodes abrocomae	*Abrothrix longipilis*	Guglielmone, 2010
Ixodes abrocomae	*Abrothrix olivaceus*	Guglielmone, 2010
Ixodes abrocomae	*Phyllotis xanthopygus*	Guglielmone, 2010
Ixodes sigelos	*Abrocoma bennetti*	González-Acuña, 2005
Ixodes sigelos	*Aconaemys fuscus*	González-Acuña, 2005
Ixodes sigelos	*Octodon bridgesi*	Muñoz-Leal 2019
Ixodes sigelos	*Octodon degus*	González-Acuña, 2005
Ixodes sigelos	*Oligoryzomys longicaudatus*	González-Acuña, 2005
Ixodes sigelos	*Phyllotis darwini*	Muñoz-Leal 2019
Ixodes sigelos	*Rattus norvegicus*	Muñoz-Leal 2019; González-Acuña, 2005
Ixodes stilesi	*Oligoryzomys longicaudatus*	Ivanova 2014
Laelaps echidninus	*Rattus norvegicus*	
Laelaps echidninus	*Rattus rattus*	Lareschi, 2010
Leptopsylla segnis	*Mus musculus*	Moreno Salas, 2020
Leptopsylla segnis	*Octodon degus*	Beaucournu, 2014; Moreno Salas, 2020
Leptopsylla segnis	*Oligoryzomys longicaudatus*	Beaucournu, 2014; Moreno Salas, 2020
Leptopsylla segnis	*Rattus norvegicus*	Beaucournu, 2014; Moreno Salas, 2020
Leptopsylla segnis	*Rattus rattus*	Moreno Salas, 2019; Moreno Salas, 2020
Leptopsylla segnis	*Abrothrix olivaceus*	Beaucournu, 2014; Moreno Salas, 2020
Listronius fortis	*Abrothrix longipilis*	Beaucournu, 2014
Listronius fortis	*Abrothrix olivaceus*	Beaucournu, 2014
Listronius plesiomorphus	*Abrothrix longipilis*	Beaucournu, 2014
Listronius plesiomorphus	*Chelemys macronyx*	Beaucournu, 2014
Listronius ulus	*Abrothrix longipilis*	Beaucournu, 2014

Listronius ulus	*Abrothrix olivaceus*	Beaucournu, 2014
Listronius ulus	*Loxodontomys micropus*	Beaucournu, 2014
Listronius ulus	*Reithrodon auritus*	Beaucournu, 2014
Lukoschus maresi	*Abrothrix olivaceus*	Lareschi, 2010
Monogyropus longus	*Abrocoma bennetti*	Ewing, 1924; Yáñez-Meza, 2018
Mysolaelaps microspinosus	*Oligoryzomys longicaudatus*	Lareschi, 2010
Neotyphloceras chilensis	*Abrothrix longipilis*	Beaucournu, 2014; Moreno Salas, 2020
Neotyphloceras chilensis	*Abrocoma bennetti*	Beaucournu, 2014; Moreno Salas, 2020; Yáñez-Meza, 2018
Neotyphloceras chilensis	*Octodon bridgesi*	Beaucournu, 2014
Neotyphloceras chilensis	*Octodon degus*	Beaucournu, 2014; Moreno Salas, 2020
Neotyphloceras chilensis	*Oligoryzomys longicaudatus*	Beaucournu, 2014; Moreno Salas, 2020
Neotyphloceras chilensis	*Phyllotis darwini*	Beaucournu, 2014; Moreno Salas, 2020
Neotyphloceras chilensis	*Phyllotis xanthopygus*	Beaucournu, 2014
Neotyphloceras chilensis	*Rattus rattus*	Beaucournu, 2014; Moreno Salas, 2019; Moreno Salas, 2020
Neotyphloceras chilensis	*Abrothrix olivaceus*	Beaucournu, 2014; Moreno Salas, 2020
Neotyphloceras crassispina	*Abrocoma cinerea*	Beaucournu, 2014
Neotyphloceras crassispina	*Abrothrix andinus*	Beaucournu, 2014
Neotyphloceras crassispina	*Abrothrix longipilis*	Beaucournu, 2014; Moreno Salas, 2020
Neotyphloceras crassispina	*Aconaemys porteri*	Beaucournu, 2014
Neotyphloceras crassispina	*Akodon albiventer*	Beaucournu, 2014
Neotyphloceras crassispina	*Chelemys macronyx*	Beaucournu, 2014
Neotyphloceras crassispina	*Ctenomys opimus*	Beaucournu, 2014
Neotyphloceras crassispina	*Loxodontomys micropus*	Beaucournu, 2014
Neotyphloceras crassispina	*Octodon degus*	Beaucournu, 2014
Neotyphloceras crassispina	*Octodontomys gliroides*	Beaucournu, 2014
Neotyphloceras crassispina	*Oligoryzomys longicaudatus*	Beaucournu, 2014; Moreno Salas, 2020
Neotyphloceras crassispina	*Phyllotis darwini*	Beaucournu, 2014; Moreno Salas, 2020
Neotyphloceras crassispina	*Phyllotis xanthopygus*	Beaucournu, 2014
Neotyphloceras crassispina	*Rattus rattus*	Beaucournu, 2014
Neotyphloceras crassispina	*Reithrodon auritus*	Beaucournu, 2014
Neotyphloceras crassispina	*Abrothrix olivaceus*	Beaucournu, 2014; Moreno Salas, 2020
Neotyphloceras pardinasi	*Abrothrix olivaceus*	Moreno Salas, 2019; Moreno Salas, 2020
Neotyphloceras pardinasi	*Rattus rattus*	Moreno Salas, 2019; Moreno Salas, 2020
Neotyphloceras pardinasi	*Abrothrix longipilis*	Moreno Salas, 2019; Moreno Salas, 2020

Neotyphloceras pardinasi	*Oligoryzomys longicaudatus*	Moreno Salas, 2019; Moreno Salas, 2020
Nonnapsylla rothschildi	*Abrocoma cinerea*	Beaucournu, 2014
Nonnapsylla rothschildi	*Octodontomys gliroides*	Beaucournu, 2014
Nosopsyllus fasciatus	*Abrothrix longipilis*	Beaucournu, 2014; Moreno Salas, 2020
Nosopsyllus fasciatus	*Abrothrix olivaceus*	Beaucournu, 2014; Moreno Salas, 2020
Nosopsyllus fasciatus	*Aconaemys porteri*	Beaucournu, 2014; Moreno Salas, 2020
Nosopsyllus fasciatus	*Myocastor coypus*	Beaucournu, 2014; Moreno Salas, 2020
Nosopsyllus fasciatus	*Oligoryzomys longicaudatus*	Beaucournu, 2014; Moreno Salas, 2020
Nosopsyllus fasciatus	*Rattus rattus*	Beaucournu, 2014; Moreno Salas, 2019; Moreno Salas, 2020; Seguel, 2017
Ornithodoros octodontus	*Octodon degus*	Muñoz-Leal 2020
Ornithonyssus bacoti	*Rattus rattus*	Seguel, 2017
Paraguacarus santiagoensis	*Octodon degus*	Goff, 1989
Paratrombicula enciscoensis	*Abrothrix olivaceus*	Silva de la Fuente, 2021
Paratrombicula goffi	*Abrothrix olivaceus*	Silva de la Fuente, 2023; Silva de la Fuente, 2021
Paratrombicula goffi	*Geoxus valdivianus*	Silva de la Fuente, 2023
Paratrombicula goffi	*Oligoryzomys longicaudatus*	Silva de la Fuente, 2023
Paratrombicula neuquenensis	*Abrothrix manni*	Silva de la Fuente, 2023
Paratrombicula neuquenensis	*Abrothrix olivaceus*	Silva de la Fuente, 2023
Paratrombicula neuquenensis	*Oligoryzomys longicaudatus*	Silva de la Fuente, 2023
Phtheiropoios nematophallus	*Ctenomys opimus*	Moreno Salas, 2005
Phtheiropoios pearsoni	*Abrocoma bennetti*	Moreno Salas, 2005
Phtheiropoios pollicaris	*Ctenomys magellanicus*	Moreno Salas, 2005
Plocopsylla achilles	*Abrothrix longipilis*	Beaucournu, 2014
Plocopsylla achilles	*Rattus rattus*	Beaucournu, 2014; Moreno Salas, 2020
Plocopsylla consobrina	*Abrothrix longipilis*	Beaucournu, 2014
Plocopsylla crypta	*Abrothrix olivaceus*	Beaucournu, 2014
Plocopsylla crypta	*Octodon degus*	Beaucournu, 2014
Plocopsylla crypta	*Phyllotis darwini*	Beaucournu, 2014
Plocopsylla diana	*Abrothrix longipilis*	Beaucournu, 2014
Plocopsylla diana	*Abrothrix olivaceus*	Beaucournu, 2014
Plocopsylla diana	*Aconaemys porteri*	Beaucournu, 2014
Plocopsylla enderleini	*Chinchillula sahamae*	Beaucournu, 2014
Plocopsylla fuegina	*Ctenomys magellanicus*	Beaucournu, 2014
Plocopsylla lewisi	*Abrothrix longipilis*	Beaucournu, 2014
Plocopsylla lewisi	*Abrothrix olivaceus*	Beaucournu, 2014
Plocopsylla lewisi	*Euneomys chinchilloides*	Beaucournu, 2014
Plocopsylla lewisi	*Oligoryzomys longicaudatus*	Beaucournu, 2014
Plocopsylla lewisi	*Reithrodon auritus*	Beaucournu, 2014

Plocopsylla muruai	*Abrothrix longipilis*	Beaucournu, 2014
Plocopsylla muruai	*Abrothrix olivaceus*	Beaucournu, 2014
Plocopsylla reigi	*Abrothrix olivaceus*	Beaucournu, 2014
Plocopsylla reigi	*Chelemys macronyx*	Beaucournu, 2014
Plocopsylla reigi	*Geoxus valdivianus*	Beaucournu, 2014
Plocopsylla reigi	*Oligoryzomys longicaudatus*	Beaucournu, 2014
Plocopsylla reigi	*Phyllotis darwini*	Beaucournu, 2014
Plocopsylla silewi	*Abrothrix olivaceus*	Sanchez, 2015; Beaucournu, 2014
Plocopsylla viracocha	*Andinomys edax*	Beaucournu, 2014
Plocopsylla viracocha	*Auliscomys boliviensis*	Beaucournu, 2014
Plocopsylla viracocha	*Chinchillula sahamae*	Beaucournu, 2014
Plocopsylla viracocha	*Phyllotis darwini*	Beaucournu, 2014
Plocopsylla viracocha	*Phyllotis osgoodi*	Beaucournu, 2014
Plocopsylla wilesi	*Abrothrix olivaceus*	Sanchez 2015; Beaucournu, 2014
Plocopsylla wilesi	*Phyllotis xanthopygus*	Beaucournu, 2014
Plocopsylla wolffsohni	*Abrothrix longipilis*	Beaucournu, 2014; Moreno Salas, 2019; Moreno Salas, 2020
Plocopsylla wolffsohni	*Abrothrix olivaceus*	Beaucournu, 2014; Moreno Salas, 2019; Moreno Salas, 2020
Plocopsylla wolffsohni	*Chelemys macronyx*	Beaucournu, 2014; Moreno Salas, 2019; Moreno Salas, 2020
Plocopsylla wolffsohni	*Octodon degus*	Beaucournu, 2014; Moreno Salas, 2019; Moreno Salas, 2020
Plocopsylla wolffsohni	*Phyllotis darwini*	Beaucournu, 2014; Moreno Salas, 2019; Moreno Salas, 2020
Plocopsylla wolffsohni	*Rattus rattus*	Beaucournu, 2014; Moreno Salas, 2019; Moreno Salas, 2020
Poliremotus chilensis	*Octodon degus*	Brennan, 1978
Polygenis platensis	*Ctenomys fulvus*	Beaucournu, 2014
Polygenis platensis	*Ctenomys robustus*	Beaucournu, 2014
Pulex irritans	*Lagidium viscacia*	Macchiavello, 1976; Ruiz del Rio, 1939
Pulex irritans	*Mus musculus*	Macchiavello, 1976; Ruiz del Rio, 1939
Pulex irritans	*Rattus norvegicus*	Macchiavello, 1976; Ruiz del Rio, 1939
Pulex irritans	*Rattus rattus*	Macchiavello, 1976; Ruiz del Rio, 1939
Quadraseta chiloensis	*Abrothrix manni*	Silva de la Fuente, 2023
Quadraseta chiloensis	*Abrothrix olivaceus*	Silva de la Fuente, 2021; Silva de la Fuente, 2023
Quadraseta chiloensis	*Geoxus valdivianus*	Silva de la Fuente, 2021; Silva de la Fuente, 2023
Quadraseta chiloensis	*Oligoryzomys longicaudatus*	Silva de la Fuente, 2021; Silva de la Fuente, 2023
Rhipicephalus sanguineus	*Rattus norvegicus*	González-Acuña , 2005
Sphinctopsylla ares	*Abrothrix lanosus*	Beaucournu, 2014; Moreno Salas, 2019; Moreno Salas, 2020

Sphinctopsylla ares	*Abrothrix longipilis*	Beaucournu, 2014; Moreno Salas, 2019; Moreno Salas, 2020
Sphinctopsylla ares	*Abrothrix olivaceus*	Beaucournu, 2014; Moreno Salas, 2019; Moreno Salas, 2020
Sphinctopsylla ares	*Abrothrix sanborni*	Beaucournu, 2014; Moreno Salas, 2019; Moreno Salas, 2020
Sphinctopsylla ares	*Aconaemys porteri*	Beaucournu, 2014; Moreno Salas, 2019; Moreno Salas, 2020
Sphinctopsylla ares	*Chelemys macronyx*	Beaucournu, 2014; Moreno Salas, 2019; Moreno Salas, 2020
Sphinctopsylla ares	*Euneomys mordax*	Beaucournu, 2014; Moreno Salas, 2019; Moreno Salas, 2020
Sphinctopsylla ares	*Geoxus valdivianus*	Beaucournu, 2014; Moreno Salas, 2019; Moreno Salas, 2020
Sphinctopsylla ares	*Loxodontomys micropus*	Beaucournu, 2014; Moreno Salas, 2019; Moreno Salas, 2020
Sphinctopsylla ares	*Loxodontomys pikumche*	Beaucournu, 2014; Moreno Salas, 2019; Moreno Salas, 2020
Sphinctopsylla ares	*Oligoryzomys longicaudatus*	Beaucournu, 2014; Moreno Salas, 2019; Moreno Salas, 2020
Sphinctopsylla ares	*Phyllotis darwini*	Beaucournu, 2014; Moreno Salas, 2019; Moreno Salas, 2020
Sphinctopsylla ares	*Phyllotis xanthopygus*	Beaucournu, 2014; Moreno Salas, 2019; Moreno Salas, 2020
Sphinctopsylla ares	*Rattus rattus*	Beaucournu, 2014; Moreno Salas, 2019; Moreno Salas, 2020
Sphinctopsylla ares	*Reithrodon auritus*	Beaucournu, 2014; Moreno Salas, 2019; Moreno Salas, 2020
Sphinctopsylla ares	*Spalacopus cyanus*	Beaucournu, 2014; Moreno Salas, 2019; Moreno Salas, 2020
Tetrapsylllus corfidii	*Octodon degus*	Moreno Salas, 2020
Tetrapsyllus amplus	*Abrothrix olivaceus*	Beaucournu, 2014; Moreno Salas, 2020
Tetrapsyllus amplus	*Abrothrix longipilis*	Beaucournu, 2014; Moreno Salas, 2020
Tetrapsyllus amplus	*Oligoryzomys longicaudatus*	Beaucournu, 2014; Moreno Salas, 2020
Tetrapsyllus amplus	*Phyllotis darwini*	Beaucournu, 2014; Moreno Salas, 2020
Tetrapsyllus bleptus	*Phyllotis darwini*	Beaucournu, 2014
Tetrapsyllus bleptus	*Phyllotis magister*	Beaucournu, 2014
Tetrapsyllus bleptus	*Phyllotis xanthopygus*	Beaucournu, 2014
Tetrapsyllus comis	*Octodon bridgesi*	Beaucournu, 2014
Tetrapsyllus contortrix	*Abrothrix olivaceus*	Beaucournu, 2014
Tetrapsyllus contortrix	*Phyllotis darwini*	Beaucournu, 2014
Tetrapsyllus corfidii	*Abrocoma bennetti*	Beaucournu, 2014; Moreno Salas, 2020; Yáñez-Meza, 2018

Tetrapsyllus corfidii	*Abrothrix longipilis*	Beaucournu, 2014; Moreno Salas, 2020
Tetrapsyllus corfidii	*Abrothrix olivaceus*	Beaucournu, 2014; Moreno Salas, 2020
Tetrapsyllus corfidii	*Aconaemys fuscus*	Beaucournu, 2014; Moreno Salas, 2020
Tetrapsyllus corfidii	*Octodon bridgesi*	Beaucournu, 2014; Moreno Salas, 2020
Tetrapsyllus corfidii	*Octodon degus*	Beaucournu, 2014; Moreno Salas, 2020
Tetrapsyllus corfidii	*Octodon lunatus*	Beaucournu, 2014; Moreno Salas, 2020
Tetrapsyllus corfidii	*Phyllotis darwini*	Beaucournu, 2014; Moreno Salas, 2020
Tetrapsyllus elutus	*Auliscomys sublimis*	Beaucournu, 2014
Tetrapsyllus elutus	*Phyllotis xanthopygus*	Beaucournu, 2014
Tetrapsyllus maulinus	*Ctenomys colburni*	Beaucournu, 2014
Tetrapsyllus maulinus	*Ctenomys magellanicus*	Beaucournu, 2014
Tetrapsyllus maulinus	*Ctenomys maulinus*	Beaucournu, 2014
Tetrapsyllus rhombus	*Abrothrix longipilis*	Beaucournu, 2014; Moreno Salas, 2019; Moreno Salas, 2020
Tetrapsyllus rhombus	*Oligoryzomys longicaudatus*	Beaucournu, 2014; Moreno Salas, 2019; Moreno Salas, 2020
Tetrapsyllus rhombus	*Phyllotis darwini*	Beaucournu, 2014; Moreno Salas, 2019; Moreno Salas, 2020
Tetrapsyllus rhombus	*Rattus rattus*	Beaucournu, 2014; Moreno Salas, 2019; Moreno Salas, 2020
Tetrapsyllus rhombus	*Abrothrix olivaceus*	Beaucournu, 2014; Moreno Salas, 2019; Moreno Salas, 2020
Tetrapsyllus rhombus	*Aconaemys porteri*	Beaucournu, 2014; Moreno Salas, 2019; Moreno Salas, 2020
Tetrapsyllus rhombus	*Ctenomys maulinus*	Beaucournu, 2014; Moreno Salas, 2019; Moreno Salas, 2020
Tetrapsyllus rhombus	*Euneomys mordax*	Beaucournu, 2014; Moreno Salas, 2019; Moreno Salas, 2020
Tetrapsyllus rhombus	*Geoxus valdivianus*	Beaucournu, 2014; Moreno Salas, 2019; Moreno Salas, 2020
Tetrapsyllus rhombus	*Loxodontomys micropus*	Beaucournu, 2014; Moreno Salas, 2019; Moreno Salas, 2020
Tetrapsyllus rhombus	*Loxodontomys pikumche*	Beaucournu, 2014; Moreno Salas, 2019; Moreno Salas, 2020
Tetrapsyllus rhombus	*Rattus norvegicus*	Beaucournu, 2014; Moreno Salas, 2019; Moreno Salas, 2020
Tetrapsyllus rhombus	*Reithrodon auritus*	Beaucournu, 2014; Moreno Salas, 2019; Moreno Salas, 2020
Tetrapsyllus satyrus	*Aconaemys fuscus*	Beaucournu, 2014
Tetrapsyllus satyrus	*Aconaemys porteri*	Beaucournu, 2014
Tetrapsyllus satyrus	*Chelemys macronyx*	Beaucournu, 2014

Tetrapsyllus satyrus	*Geoxus valdivianus*	Beaucournu, 2014
Tetrapsyllus simulans	*Abrothrix longipilis*	Beaucournu, 2014
Tetrapsyllus simulans	*Oligoryzomys longicaudatus*	Beaucournu, 2014
Tetrapsyllus simulans	*Phyllotis darwini*	Beaucournu, 2014
Tetrapsyllus tantillus	*Abrocoma bennetti*	Beaucournu, 2014; Moreno Salas, 2020
Tetrapsyllus tantillus	*Abrothrix olivaceus*	Beaucournu, 2014; Moreno Salas, 2020
Tetrapsyllus tantillus	*Ctenomys magellanicus*	Beaucournu, 2014; Moreno Salas, 2020
Tetrapsyllus tantillus	*Euneomys mordax*	Beaucournu, 2014; Moreno Salas, 2020
Tetrapsyllus tantillus	*Loxodontomys micropus*	Beaucournu, 2014; Moreno Salas, 2020
Tetrapsyllus tantillus	*Octodon degus*	Beaucournu, 2014; Moreno Salas, 2020
Tetrapsyllus tantillus	*Phyllotis darwini*	Beaucournu, 2014; Moreno Salas, 2020
Tetrapsyllus tantillus	*Phyllotis xanthopygus*	Beaucournu, 2014; Moreno Salas, 2020
Tetrapsyllus tantillus	*Reithrodon auritus*	Beaucournu, 2014; Moreno Salas, 2020
Tetrapsyllus tantillus	*Spalacopus cyanus*	Beaucournu, 2014; Moreno Salas, 2020
Tetrapsyllus tantillus	*Abrothrix longipilis*	Beaucournu, 2014; Moreno Salas, 2020
Tiamastus callens	*Ctenomys maulinus*	Beaucournu, 2014
Tiamastus gallardoi	*Abrothrix olivaceus*	Beaucournu, 2014
Tiamastus gallardoi	*Ctenomys colburni*	Beaucournu, 2014
Tiamastus plesius	*Ctenomys robustus*	Beaucournu, 2014
Tunga bonneti	*Phyllotis darwini*	Beaucournu, 2014
Tunga bonneti	*Phyllotis xanthopygus*	Beaucournu, 2014
Tunga bonneti	*Rattus rattus*	Beaucournu, 2014
Tunga libis	*Phyllotis darwini*	Beaucournu, 2014
Tunga penetrans	*Rattus norvegicus*	Beaucournu, 2014
Xenopsylla astia	*Rattus norvegicus*	Beaucournu, 2014
Xenopsylla cheopis	*Rattus norvegicus*	Beaucournu, 2014
Xenopsylla cheopis	*Rattus rattus*	Beaucournu, 2014; Moreno Salas, 2019; Moreno Salas, 2020
**Author, Year**	**Reference**	
Beaucournu, 2014	Beaucournu, J.-C., & González-Acuña, D. (2014). Fleas (Insecta-Siphonaptera) of Chile: A review. *Zootaxa*, 3900, 151-203. https://doi.org/10.11646/zootaxa.3900.2.1	
Brennan, 1978	Brennan, J. M., & Goff, M. L. (1978). Three new monotypic genera of chiggers (Acari: Trombiculidae) from South America. J Med Entomol, 14(5), 541-544. https://doi.org/10.1093/jmedent/14.5.541	

Castro, 1981	Castro, D. C. (1981). Contribución al conocimiento de los Anoplura neotropicales. *Revista de la Sociedad de Entomología de Argentina*, *40*, 231-236.	
Castro, 2002	Castro, D., & Cicchino, A. (2002). Las especies del género Gyropus Nitzsch, 1818 (Phthiraptera: Gyropidae) parásitas de Octodontidae (Mammalia: Rodentia). *Revista Chilena de Historia Natural*, 75. https://doi.org/10.4067/S0716-078X2002000200003	
Durden, 2000	Durden, L. A., & Gomez, M. S. (2000). Abrocomaphthirus chilensis (Gomez), new combination (Phthiraptera-Anoplura), an ectoparasite of the Chilean rodent Abrocoma bennetti (Abrocomidae). *Parasites*, 7(4), 331-332. https://doi.org/10.1051/parasite/2000074331	
Emerson, 1976	Emerson, K. C., & Price, R. D. (1976). Abrocomophagidae (Mallophaga: Amblycera), a New Family from Chile. *The Florida Entomologist*, 59(4), 425-428. https://doi.org/10.2307/3494196	
Ewing, 1924	Ewing, H. E. (1924). On the taxonomy, biology, and distribution of the biting lice of the family Gyropidae. *Proceedings of the United States National Museum*, 63, 1-42. https://doi.org/ https://doi.org/10.5479/si.00963801.63-2489.1	
Fuenzalida-Araya, 2022	Fuenzalida-Araya, K., González-Aguayo, F., Moreno, L., Landaeta-Aqueveque, C., Santodomingo, A., Silva-de la Fuente, C., & González-Acuña, D. (2022). New records of Gigantolaelaps wolffsohni (Mesostigmata: Laelapidae) in Chile, an ectoparasite of Oligoryzomys longicaudatus (Rodentia: Cricetidae): ecological aspects and relation to body size and sex of their host. *Acaralogia*, 62(4), 965-973. https://doi.org/10.24349/nze0-ju8m	
Goff, 1989	Goff, M. L., & Webb, J. P. (1989). A new species of *Paraguacarus* (Acari: Trombiculidae) from a degu (Mammalia: Rodentia) collected in Chile. *Bulletin of the Society for Vector Ecology*, *14*(1), 93-94.	

Gomez, 1998	Gomez, M. S. (1998). Two Anoplura species from rodents in Chile: Hoplopleura andina Castro, 1981 (Hoplopleuridae) from Geoxus valdivianus (Cricetidae) and Eulinognathus chilensis n. sp. (Polyplacidae) from Abracoma bennetti (Abracomidae). *Research and Reviews in Parasitology*, *58*(1), 49-54.	
González-Acuña, 2003	González-Acuña, D., Castro, D. d. C., & Moreno-Salas, L. (2003). Contribucion al Conocimiento de los *Phthiraptera* (Anoplura, Hoplopleura) Parasitos de Roedores en Chile. *Gayana (Concepción)*, 67(1), 118-120. https://dx.doi.org/10.4067/S0717-65382003000100014	
González-Acuña, 2005	González-Acuña, D., Castro, D. d. C., Moreno Salas, L. d. C., Torres-Mura, J. C., & Mey, E. (2005). New records of Sucking lice (Insecta: Phthiraptera: Anoplura) on rodents (Mammalia: Rodentia: Muridae) from Chile. *Mastozoologia Neotropical*. https://www.biodiversitylibrary.org/part/113485	
González-Acuña, 2005	González-Acuña, D., & Guglielmone, A. A. (2005). Ticks (Acari: Ixodoidea: Argasidae, Ixodidae) of Chile. *Experimental Applied Acarology*, 35(1-2), 147-163. https://doi.org/10.1007/s10493-004-1988-2	
Guglielmone, 2010	Guglielmone, A. A., Nava, S., Bazan-Leon, E. A., Vasquez, R. A., & Mangold, A. J. (2010). Redescription of the male and description of the female of Ixodes abrocomae Lahille, 1916 (Acari: Ixodidae). *Systematic Parasitology*, 77(2), 153-160. https://doi.org/10.1007/s11230-010-9262-y	
Ivanova, 2014	Ivanova, L. B., Tomova, A., González-Acuña, D., Murúa, R., Moreno, C. X., Hernández, C., Cabello, J., Cabello, C., Daniels, T. J., Godfrey, H. P., & Cabello, F. C. (2014). Borrelia chilensis, a new member of the Borrelia burgdorferi sensu lato complex that extends the range of this genospecies in the Southern Hemisphere. *Environmental Microbiology*, 16(4), 1069-1080. https://doi.org/10.1111/1462-2920.12310	
Macchiavello, 1948	Macchiavello, A. S. (1948). Siphonaptera de la costa Sur-Occidental de América (Primera lista y distribucion zoo-geográfica). *Boletin de la Oficina Sanitaria Panamericana*, *27*.	
Moreno-Salas, 2005	Moreno-Salas, L., del C. Castro, D., Torres-Mura, J. C., & Gonzalez-Acuna, D. (2005). Phthiraptera (Amblycera and Anoplura) parasites of the family Octodontidae, Ctenomyidae and Abrocomidae (Mammalia: Rodentia) from Chile. *Rudolstaedter Naturhistorische Schriften*, *13*, 115-118.	

Moreno-Salas, 2019	Moreno-Salas, L., Espinoza-Carniglia, M., Lizama Schmeisser, N., Torres, L. G., Silva-de la Fuente, M. C., Lareschi, M., & González-Acuña, D. (2019). Fleas of black rats (Rattus rattus) as reservoir host of Bartonella spp. in Chile. *PeerJ*, 7, e7371. https://doi.org/10.7717/peerj.7371	
Moreno-Salas, 2020	Moreno-Salas, L., Espinoza-Carniglia, M., Lizama-Schmeisser, N., Torres-Fuentes, L. G., Silva-de La Fuente, M. C., Lareschi, M., & González-Acuña, D. (2020). Molecular detection of Rickettsia in fleas from micromammals in Chile. *Parasites and Vectors*, 13(1), 523. https://doi.org/10.1186/s13071-020-04388-5	
Muñoz-Leal, 2019	Muñoz-Leal S, Marcili, A., Fuentes-Castillo, D., Ayala, M., & Labruna, M. B. (2019). A relapsing fever *Borrelia* and spotted fever *Rickettsia* in ticks from an Andean valley, central Chile. *Experimental and Applied Acarology*, 78(3), 403-420. https://doi.org/10.1007/s10493-019-00389-x.	
Muñoz-Leal, 2020	Muñoz-Leal, S., Venzal, J. M., Nava, S., Marcili, A., Gonzalez-Acuna, D., Martins, T. F., & Labruna, M. B. (2020). Description of a new soft tick species (Atari: Argasidae: Ornithodoros) parasite of Octodon degus (Rodentia: Octodontidae) in northern Chile. *Ticks And Tick-Borne Diseases*, 11(3). https://doi.org/10.1016/j.ttbdis.2020.101385	
Price, 2000	Price, R. D., & Timm, R. M. (2000). Review of the chewing louse genus Abrocomophaga (Phthiraptera: Amblycera), with description of two new species. *Proceedings of the Biological Society of Washington*, *113*(1), 210-217.	
Ruiz del Rio, 1939	Ruiz del Rio, A. (1939). Contribution al estudio de las enfermedades parasitarias humanas transmitidas por las ratas en Concepcion. *Boletin de la Sociedad de Biologia de Concepcion*, *13*(no. 1), pp. 47-76	
Sanchez, 2015	Sanchez, J., Beaucournu, J. C., & Lareschi, M. (2015). Revision of fleas of the genus *Plocopsylla* belonging to the 'angusticeps-lewisi' complex in the Andean biogeographic region, with the description of a new species. *Medical and Veterinary Entomology*, *29*(2), 147-158. https://doi.org/10.1111/mve.12105	

Seguel, 2017	Seguel, M., Muñoz, F., Paredes, E., Navarrete, M. J., & Gottdenker, N. L. (2017). Pathological Findings in Wild Rats (Rattus rattus) Captured at Guafo Island, Northern Chilean Patagonia. *Journal of Comparative Pathology*, *157*(2-3), 163-173. https://doi.org/10.1016/j.jcpa.2017.07.006	
Sikora, 2012	Sikora, B., & Bochkov, A. V. (2012). Fur mites of the family Listrophoridae (Acariformes: Sarcoptoidea) associated with South American sigmodontine rodents (Cricetidae: Sigmodontinae). *Acta Parasitologica*, 57(4), 388-396. https://doi.org/10.2478/s11686-012-0046-1	
Silva de la Fuente, 2016	Silva de la Fuente, M. C., Casanueva, M. E., Salas, L. M., & González-Acuña, D. (2016). A new genus and species of chigger mite (Trombidiformes: Trombiculidae) from Loxodontomys pikumche (Rodentia: Cricetidae) in Chile. *Zootaxa*, 4092(3), 426-430. https://doi.org/10.11646/zootaxa.4092.3.8	
Silva de la Fuente, 2021	Silva-de La Fuente, M. C., Stekolnikov, A. A., Weitzel, T., Beltrami, E., Martinez-Valdebenito, C., Abarca, K., & Acosta-Jamett, G. (2021). Chigger Mites (Acariformes: Trombiculidae) of Chiloe Island, Chile, With Descriptions of Two New Species and New Data on the Genus Herpetacarus. *Journal of Medical Entomology*, 58(2), 646-657. https://doi.org/10.1093/jme/tjaa258	
Silva de la Fuente, 2023	Silva de la Fuente, M. C., Perez, C., Martinez-Valdebenito, C., Perez, R., Vial, C., Stekolnikov, A., Abarca, K., Weitzel, T., & Acosta-Jamett, G. (2023). Eco-epidemiology of rodent-associated trombiculid mites and infection with *Orientia spp*. in Southern Chile. *Plos Neglected Tropical Diseases*, *17*(1), e0011051-e0011051. https://doi.org/10.1371/journal.pntd.0011051	
Silva-de la Fuente, 2020	Silva-de la Fuente MC, Moreno Salas L, Casanueva ME, Lareschi M, González-Acuña D. (2020). Morphometric variation of *Androlaelaps fahrenholzi* (Mesostigmata: Laelapidae) associated with three Sigmodontinae (Rodentia: Cricetidae) from the north of Chile. *Experimental and Applied Acarology*. 81**:** 135-48. doi: 10.1007/s10493-020-00490-6	

Veloso-Frias, 2019	Veloso-Frias, J., Silva-De La Fuente, M. C., Rubio, A. V., Moreno, L., Gonzalez-Acuna, D., Simonetti, J. A., & Landaeta-Aqueveque, C. (2019). Variation in the prevalence and abundance of mites parasitizing Abrothrix olivacea (Rodentia) in the native forest and Pinus radiata plantations in central Chile. *Hystrix-Italian Journal Of Mammalogy*, 30(2), 107-111. https://doi.org/10.4404/hystrix-00171-2019	
Webb, 1986	Webb, J. P., Jr., Bennett, S. G., & Loomis, R. B. (1986). A new genus and species of trombiculid mite (Acari) from a Chilean rodent (Mammalia: Cricetidae). *International Journal of Acarology*, *12*(2), 83-85.	
Yanez-Meza, 2019	Yanez-Meza, A., Landacta-Aqueveque, C., Quiroga, N., & Botto-Mahan, C. (2019). Helminthic infection in three native rodent species from a semiarid Mediterranean ecosystem. *Revista Brasileira de Parasitologia Veterinaria*, 28(1), 119-125.	

Table S2. Name changes made according to the Integrated Taxonomic Information System

Rodent Species Name in Review Literature	Valid Name according to ITIS
Abrothrix olivacea	*Abrothrix olivaceus*
Abrothrix xanthorhinus	*Abrothrix olivaceus*
Abrothrix brachiotis	*Abrothrix olivaceus*
Abrothrix hirta	*Abrothrix longipilis*
Reithrodon physodes	*Reithrodon auritus*
Akodon berlepschii	*Akodon albiventer*
Phyllotis xanthorhinus	*Phyllotis xanthropygus*

Table S3. Data summary for predictor variables: The total ectoparasite associations and relationships for all rodents.

Supplemental Table S3		
Rodent	Total Parasites	Total Relationships
Abrocoma bennettii	18	59
Abrocoma cinerea	2	16
Abrothrix andinus	2	17
Abrothrix lanosus	1	15
Abrothrix longipilis	34	146
Abrothrix manni	4	12
Abrothrix olivaceus	46	184
Abrothrix sanborni	5	38

Aconaemys fuscus	8	26
Aconaemys porteri	9	66
Aconaemys sagei	1	5
Akodon albiventer	3	19
Andinomys edax	3	5
Auliscomys boliviensis	1	4
Auliscomys sublimis	1	1
Cavia porcellus	1	1
Chelemys macronyx	8	54
Chinchillula sahamae	2	4
Ctenomys colburni	2	3
Ctenomys fulvus	2	1
Ctenomys magellanicus	4	12
Ctenomys maulinus	4	14
Ctenomys opimus	2	15
Ctenomys osgoodi	1	0
Ctenomys robustus	3	4
Eligmodontia puerulus	2	1
Eligmodontia typus	1	5
Euneomys chinchilloides	4	4
Euneomys mordax	4	42
Geoxus valdivianus	13	65
Irenomys tarsalis	1	6
Lagidium viscacia	3	3
Loxodontomys micropus	15	93
Loxodontomys pikumche	3	27
Mus musculus	2	8
Myocastor coypus	1	5
Octodon bridgesi	5	24
Octodon degus	22	79
Octodon lunatus	4	13
Octodontomys gliroides	6	20
Octomys mimax	1	1
Oligoryzomys longicaudatus	29	133
Phyllotis darwini	27	127
Phyllotis magister	1	2
Phyllotis osgoodi	1	4
Phyllotis xanthopygus	15	79
Rattus norvegicus	14	33

Rattus rattus	21	101
Reithrodon auritus	9	72
Spalacopus cyanus	5	36

Table S4. Logistic regression coefficients for ectoparasite sharing between rodents based on predictor variables: The logistic regression coefficients (slope, intercept, and their corresponding measures such as z-value, p-value, standard error, and confidence intervals) are reported for each ectoparasite species included in the analysis of predictor variables. The three variables included are geographic overlap, geographic distance, and phylogenetic relatedness. The coefficients for hantavirus-sharing and the entire dataset with and without hantavirus included are reported.

Geographic Overlap	intercept	Std. Error	z value	Pr(>|z|)	2.50%	97.50%	slope	Std. Error	z value	Pr(>|z|)	2.50%	97.50%
Ixodes abrocomae	-3.37984	0.897431	-3.7771	0.00017813	-5.13877	-1.6209	3.479909	1.679927	2.069796	0.041216162	0.18731156	6.772506
Ixodes sigelos	-2.5007	0.654331	-3.81892	0.00014468	-3.78316	-1.21823	2.559303	1.214298	2.085888	0.060624153	0.17932222	4.939283
Androlaelaps farenholzi	-2.76032	0.711698	-3.88126	0.00010814	-4.15522	-1.36541	2.417411	1.423384	1.688058	0.130747013	-0.3723703	5.207193
Argentinacarus expansus	-5.47106	2.700794	-2.84951	0.04782584	-10.7645	-0.1776	5.970456	3.328291	2.144289	0.063105615	-0.5528743	12.49379
Herpetacarus eloisae	-3.2276	0.869101	-3.74251	0.00025749	-4.93101	-1.52419	3.264389	1.412565	2.310294	0.021471204	0.49581305	6.032965
Quadraseta chiloensis	-5.41038	2.634019	-2.87403	0.04568993	-10.573	-0.2478	5.915841	3.266073	2.157071	0.060930005	-0.4855455	12.31723
Agastopsylla boxi	-2.90689	0.767517	-3.83926	0.00019553	-4.41119	-1.40258	3.209622	1.458805	2.194216	0.049427266	0.35041703	6.068827
Barreropsylla excelsa	-9.05301	4.422845	-2.51586	0.03317193	-17.7216	-0.38439	11.82802	5.729609	2.32048	0.033808526	0.59819132	23.05785
Chiliopsylla allophyla	-3.21817	0.842378	-3.84071	0.000147	-4.8692	-1.56714	4.372936	1.471944	2.957736	0.003815778	1.4879787	7.257894
Craneopsylla minerva	-2.9285	0.758053	-3.86727	0.00011844	-4.41426	-1.44274	2.329936	1.552345	1.521546	0.138947029	-0.712604	5.372476
Ctenoparia inopinata	-2.6149	0.665374	-3.93283	9.01E-05	-3.91901	-1.31079	3.899995	1.292304	3.010456	0.003185042	1.36712612	6.432863
Ctenoparia jordani	-8.05604	3.714359	-2.35665	0.02857907	-15.336	-0.77603	11.16373	5.18756	2.247523	0.03068968	0.99629452	21.33116
Ctenoparia topallii	-3.18767	0.834018	-3.83365	0.00014105	-4.82232	-1.55303	3.890201	1.470012	2.607718	0.013935097	1.00903075	6.771371
Delostichus coxalis	-16.5037	15.08099	-3.11146	0.06374232	-46.0619	13.05446	16.60243	15.62523	1.922613	0.103117239	-14.022462	47.22732
Delostichus phyllotis	-3.30408	0.923144	-3.59142	0.00037814	-5.1134	-1.49475	2.629128	1.462987	1.744931	0.18097306	-0.2382737	5.496529
Delostichus smiti	-3.84738	1.239126	-3.40359	0.00476531	-6.27602	-1.41873	4.115934	1.81614	2.248033	0.039189149	0.55636531	7.675503
Ectinorus chilensis	-2.98856	0.82049	-3.66312	0.00029552	-4.59669	-1.38043	2.469994	1.378119	1.753402	0.182284937	-0.2310692	5.171057
Ectinorus martini	-2.65098	0.707485	-3.75123	0.00018043	-4.03763	-1.26434	2.108717	1.146511	1.873301	0.072395872	-0.1384023	4.355836
Ectinorus onychius	-2.86928	0.767386	-3.79907	0.00026358	-4.37333	-1.36523	3.0772	1.42948	2.162366	0.064007112	0.27547082	5.878928
Hectopsylla cypha	-3.48408	0.935319	-3.74714	0.00020977	-5.31728	-1.65089	4.232196	1.720983	2.442818	0.031231519	0.85913222	7.60526
Leptopsylla segnis	-6.6644	2.625037	-3.07463	0.01080325	-11.8094	-1.51942	9.966737	3.936092	2.772539	0.011903883	2.2521379	17.68134
Listronius ulus	-4.49307	1.392217	-3.26579	0.00140567	-7.22176	-1.76437	5.396713	2.120196	2.532096	0.011920489	1.24120536	9.55222
Neotyphloceras chilensis	-2.52673	0.654763	-3.84979	0.00014964	-3.81005	-1.24342	3.707422	1.35305	2.744292	0.038410116	1.05549286	6.359351
Neotyphloceras crassispina	-0.92205	0.39353	-2.33736	0.02113462	-1.69335	-0.15075	1.480682	1.017962	1.448928	0.168229969	-0.5144867	3.475851
Neotyphloceras pardinasi	-12.4108	7.551384	-1.99137	0.0694639	-27.2112	2.389661	16.05651	9.788652	1.880244	0.077534451	-3.128892	35.24192
Nosopsyllus fasciatus	-436.275	45715.8	-2.75204	0.1891381	-90037.6	89165.04	438.4142	45716.62	2.41795	0.190886672	-89164.516	90041.34
Plocopsylla lewisi	-3.36742	0.875486	-3.86594	0.00013157	-5.08334	-1.65149	4.699046	1.810527	2.594553	0.010591529	1.15047887	8.247613
Plocopsylla reigi	-3.80911	1.081285	-3.54804	0.0005227	-5.92838	-1.68983	4.485762	1.701391	2.629164	0.008730162	1.15109605	7.820428
Plocopsylla viracocha	-3.0329	0.871332	-3.50231	0.00051108	-4.74068	-1.32512	1.973702	1.260704	1.654677	0.209094185	-0.4972319	4.444636
Plocopsylla wolffsohni	-3.32967	0.896718	-3.731	0.00023745	-5.0872	-1.57213	3.858277	1.437319	2.684783	0.007704556	1.04118237	6.675371
Pulex irritans	-4.20338	1.209992	-3.49295	0.000587	-6.57492	-1.83184	5.337801	2.128867	2.504484	0.012758424	1.16529873	9.510303
Sphinctopsylla ares	-1.13668	0.418747	-2.69353	0.01877856	-1.95741	-0.31595	2.268526	1.113685	2.089737	0.092766186	0.08574391	4.451309
Tetrapsyllus amplus	-5.34935	1.857608	-2.95189	0.00494055	-8.99019	-1.7085	6.872407	2.761788	2.501981	0.013113431	1.45940243	12.28541
Tetrapsyllus corfidii	-2.2893	0.608554	-3.7564	0.00019195	-3.48204	-1.09655	2.344557	1.126238	2.099449	0.068541062	0.13716987	4.551943
Tetrapsyllus rhombus	-1.85359	0.512299	-3.60979	0.00035623	-2.85768	-0.8495	3.495014	1.206244	2.908839	0.006064852	1.13081858	5.85921
Tetrapsyllus satyrus	-3.15868	0.89505	-3.5463	0.00044966	-4.91295	-1.40442	1.991329	1.293059	1.542682	0.133329737	-0.5430196	4.525677
Tetrapsyllus tantillus	-1.6641	0.481425	-3.44917	0.00090746	-2.60768	-0.72053	2.200837	1.160908	1.923063	0.116694131	-0.0745021	4.476175
Overall	-2.82881	0.11842	-23.8897	1.66E-124	-3.06091	-2.59671	3.076158	0.204413	15.04783	8.95E-44	2.67551514	3.4768
Hantavirus	-4.45445	1.301666	-3.43294	0.00065629	-7.00567	-1.90324	7.255885	2.292575	3.166775	0.001557015	2.76252011	11.74925
Overall including hantavirus	-2.84654	0.117543	-24.2188	1.20E-127	-3.07692	-2.61616	3.134944	0.203035	15.43817	1.63E-45	2.73700354	3.532884

Geographic distance												
Ixodes abrocomae	-1.48056	0.86201	-1.77091	0.11742897	-3.17007	0.208944	-0.10356	0.098258	-1.01708	0.319118392	-0.2961423	0.089022
Ixodes sigelos	0.138553	0.747815	0.109403	0.55411029	-1.32714	1.604243	-0.29823	0.135863	-2.15002	0.046603029	-0.564513	-0.03194
Androlaelaps farenholzi	-0.9062	0.826767	-1.16698	0.41680753	-2.52664	0.714231	-0.15127	0.100729	-1.32236	0.288411929	-0.3486937	0.046157
Argentinacarus expansus	0.304253	0.999841	0.289611	0.74587939	-1.6554	2.263906	-1.00215	0.661412	-1.50629	0.132378396	-2.2984977	0.29419
Herpetacarus eloisae	0.364411	0.902092	0.309778	0.61532653	-1.40366	2.132477	-0.59978	0.339878	-1.75859	0.082053963	-1.2659311	0.066367
Quadraseta chiloensis	0.28752	0.998466	0.273937	0.75472929	-1.66944	2.244477	-0.98606	0.651442	-1.50432	0.132907038	-2.2628612	0.290744
Agastopsylla boxi	-1.25413	0.719342	-1.70999	0.13001185	-2.66402	0.155752	-0.07085	0.069238	-0.98175	0.223032909	-0.2065526	0.064857
Barreropsylla excelsa	0.818588	0.98794	0.808411	0.44712878	-1.11774	2.754915	-1.13565	0.666189	-1.6895	0.091816899	-2.4413529	0.170059
Chiliopsylla allophyla	1.162871	0.913193	1.217802	0.2624172	-0.62695	2.952696	-0.72374	0.348758	-2.07383	0.040359318	-1.4072889	-0.04018
Craneopsylla minerva	-1.98989	0.935327	-2.14022	0.0405981	-3.82309	-0.15668	-0.02933	0.077483	-0.40413	0.695232265	-0.1811933	0.122534
Ctenoparia inopinata	0.477232	0.679874	0.685194	0.3651282	-0.8553	1.80976	-0.31727	0.137489	-2.22747	0.060231083	-0.5867421	-0.04779
Ctenoparia jordani	0.121103	0.845433	0.120737	0.83294397	-1.53592	1.778122	-0.5746	0.336505	-1.71083	0.087510729	-1.23414	0.084936
Ctenoparia topallii	-0.32334	0.718626	-0.46304	0.60021606	-1.73182	1.085145	-0.25601	0.138628	-1.78236	0.104371704	-0.5277145	0.015699
Delostichus coxalis	-0.27601	0.786325	-0.4263	0.57405804	-1.81718	1.26516	-0.29713	0.143037	-2.00323	0.063107999	-0.5774814	-0.01678
Delostichus phyllotis	-0.04294	0.885666	-0.12401	0.52145278	-1.77882	1.692932	-0.43966	0.209959	-1.99658	0.06642377	-0.8511708	-0.02815
Delostichus smiti	-0.53422	0.778411	-0.7631	0.60159468	-2.05988	0.99144	-0.2356	0.121943	-1.81208	0.105570244	-0.4746002	0.003407
Ectinorus chilensis	-0.38604	0.789938	-0.55763	0.63338836	-1.93429	1.162214	-0.27348	0.136426	-1.88813	0.093811824	-0.5408672	-0.00609
Ectinorus martini	0.840261	1.016207	0.648001	0.50397581	-1.15147	2.831991	-0.81299	0.430101	-1.88934	0.059686015	-1.6559745	0.029989
Ectinorus onychius	-1.20944	0.754984	-1.57284	0.28480521	-2.68918	0.270302	-0.06067	0.063784	-0.94745	0.16850167	-0.1856883	0.06434
Hectopsylla cypha	-1.30898	0.747747	-1.75232	0.08659934	-2.77454	0.156579	-0.09887	0.085801	-1.1337	0.288062356	-0.2670393	0.069294
Leptopsylla segnis	-0.39312	0.724608	-0.58504	0.60565987	-1.81333	1.027082	-0.23539	0.126568	-1.84321	0.068440807	-0.4834608	0.012676
Listronius ulus	-0.97669	0.77821	-1.29076	0.23967978	-2.50195	0.548577	-0.17698	0.115063	-1.55949	0.124634166	-0.402495	0.048543
Neotyphloceras chilensis	-0.14341	0.643941	-0.2743	0.57144146	-1.40551	1.118692	-0.16643	0.081915	-1.96065	0.076359686	-0.3269836	-0.00588
Neotyphloceras crassispina	-0.37292	0.54201	-0.69121	0.49576488	-1.43524	0.689402	-0.02174	0.041818	-0.49904	0.605727675	-0.1037029	0.060221
Neotyphloceras pardinasi	-0.18148	0.922328	-0.27037	0.62944011	-1.98921	1.626252	-0.57269	0.364005	-1.56799	0.116963182	-1.2861319	0.140743
Nosopsyllus fasciatus	0.325835	0.802346	0.394036	0.64499558	-1.24674	1.898405	-0.51308	0.275357	-1.88165	0.062218716	-1.052773	0.026607
Plocopsylla lewisi	-0.68078	0.776193	-1.02219	0.38714808	-2.20209	0.840527	-0.12803	0.07805	-1.65273	0.144776129	-0.2810018	0.024948
Plocopsylla reigi	-0.69253	0.7326	-0.92689	0.37697113	-2.1284	0.743345	-0.24767	0.153258	-1.57534	0.132429293	-0.5480547	0.052706
Plocopsylla viracocha	-0.57899	0.756995	-0.61237	0.72114447	-2.06267	0.904697	-0.19189	0.115187	-1.52588	0.115666049	-0.4176489	0.033876
Plocopsylla wolffsohni	-0.49862	0.694193	-0.73318	0.47687376	-1.85921	0.861976	-0.21465	0.11808	-1.818	0.069250617	-0.4460819	0.016782
Pulex irritans	-0.88553	0.87802	-1.05273	0.36095643	-2.60642	0.835359	-0.20793	0.134138	-1.51961	0.142487792	-0.4708313	0.054979
Sphinctopsylla ares	0.612354	0.58561	1.06137	0.23597554	-0.53542	1.760129	-0.13163	0.057538	-2.24796	0.062388623	-0.2444046	-0.01886
Tetrapsyllus amplus	-1.09936	0.786699	-1.38992	0.16869044	-2.64126	0.442539	-0.20077	0.14156	-1.39619	0.173092171	-0.4782176	0.076686
Tetrapsyllus corfidii	0.26506	0.711543	0.254695	0.44212803	-1.12954	1.659659	-0.27435	0.11404	-2.30844	0.036198859	-0.4978664	-0.05084
Tetrapsyllus rhombus	0.777927	0.610007	1.285665	0.21671713	-0.41766	1.97352	-0.22877	0.08477	-2.65772	0.0180977	-0.3949133	-0.06262
Tetrapsyllus satyrus	-0.4352	0.854986	-0.53704	0.61783283	-2.11094	1.240539	-0.47917	0.322713	-1.50778	0.132595056	-1.1116712	0.153341
Tetrapsyllus tantillus	-0.43943	0.618951	-0.72012	0.51718199	-1.65255	0.773688	-0.07203	0.057107	-1.25114	0.224132019	-0.1839617	0.039894
Overall	-0.64673	0.108901	-5.94198	7.21E-07	-0.86017	-0.43329	-0.15417	0.014462	-10.6306	3.77E-18	-0.1825195	-0.12583
Hantavirus	-0.17871	0.674401	-0.26365	0.77364074	-1.50052	1.143087	-0.25369	0.12987	-1.93833	0.060343221	-0.5082254	0.000854
Overall including hantavirus	-0.63713	0.107231	-5.94554	1.30E-06	-0.8473	-0.42696	-0.15559	0.014321	-10.8379	5.54E-22	-0.1836626	-0.12752

Phylogenetic Distance

Ixodes abrocomae	-1.3628	1.029219	-1.34234	0.18341107	-3.38004	0.654428	-0.02197	0.020312	-1.07236	0.307182254	-0.0617842	0.017838
Ixodes sigelos	-1.47233	0.960645	-1.49245	0.20828725	-3.35516	0.410501	-0.00475	0.01373	-0.44102	0.356901242	-0.0316566	0.022164
Androlaelaps farenholzi	-0.97866	1.02665	-0.9842	0.3803527	-2.99085	1.033541	-0.02477	0.020205	-1.18374	0.293727236	-0.0643724	0.014831
Argentinacarus expansus	9.429203	9.861618	0.563904	0.59354904	-9.89921	28.75762	-0.32796	0.278833	-1.78934	0.104412929	-0.8744624	0.218543
Herpetacarus eloisae	10.90898	8.161827	1.037609	0.31316369	-5.08791	26.90587	-0.36533	0.233779	-2.13199	0.048678009	-0.8235261	0.092872
Quadraseta chiloensis	10.11528	10.53409	0.576105	0.58575437	-10.5312	30.76172	-0.34574	0.296578	-1.77072	0.109183583	-0.9270198	0.235544
Agastopsylla boxi	0.408231	1.081818	0.176186	0.71108534	-1.71209	2.528555	-0.05486	0.028054	-1.97499	0.048849854	-0.1098437	0.000127
Barreropsylla excelsa	1.965875	4.509925	0.220359	0.70188862	-6.87342	10.80517	-0.11016	0.126102	-1.71866	0.150396376	-0.3573154	0.136996
Chiliopsylla allophyla	2.487815	3.514802	0.763408	0.45929821	-4.40107	9.376701	-0.11078	0.097528	-2.0161	0.093092272	-0.301935	0.080367
Craneopsylla minerva	0.209808	1.311729	-0.09338	0.61737296	-2.36113	2.780749	-0.06461	0.037416	-1.71379	0.087296813	-0.1379478	0.008721
Ctenoparia inopinata	-0.26286	0.829393	-0.26494	0.66886375	-1.88844	1.362724	-0.02495	0.015428	-1.5724	0.065466122	-0.0551904	0.005285
Ctenoparia jordani	-0.14182	1.064652	-0.32663	0.44951281	-2.2285	1.944857	-0.03957	0.022666	-1.73902	0.089087836	-0.0839905	0.004859
Ctenoparia topallii	-0.73693	0.891481	-0.81196	0.47260827	-2.4842	1.010336	-0.02799	0.018932	-1.3673	0.173044813	-0.0650965	0.009115
Delostichus coxalis	-1.72795	1.106022	-1.56988	0.17659745	-3.89572	0.439812	-0.00654	0.015542	-0.46214	0.44086687	-0.0370048	0.023918
Delostichus phyllotis	-1.29539	1.065664	-1.18095	0.33642451	-3.38405	0.793278	-0.02016	0.018513	-1.0893	0.16976965	-0.0564471	0.016122
Delostichus smiti	-1.83835	1.133636	-1.69822	0.15544168	-4.06023	0.383536	-0.00391	0.016033	-0.22994	0.744849837	-0.0353297	0.02752
Ectinorus chilensis	-0.78757	0.987548	-0.68647	0.46316472	-2.72313	1.147987	-0.02645	0.017951	-1.55376	0.082307379	-0.0616349	0.008733
Ectinorus martini	-1.20264	0.805097	-1.47162	0.16503167	-2.7806	0.375325	-0.01339	0.013717	-1.02203	0.370298596	-0.0402792	0.013491
Ectinorus onychius	0.461717	1.076593	0.263294	0.73108439	-1.64837	2.571801	-0.05797	0.029321	-1.9982	0.04639186	-0.1154386	-0.0005
Hectopsylla cypha	4.282969	6.804272	0.099219	0.80103755	-9.05316	17.6191	-0.17029	0.186664	-1.71854	0.12917063	-0.5361445	0.195564
Leptopsylla segnis	-0.43268	1.476431	-0.45991	0.19041487	-3.32643	2.461068	-0.0214	0.020989	-0.88106	0.304718299	-0.0625336	0.019741
Listronius ulus	0.035733	1.23319	-0.24033	0.53324878	-2.38128	2.452742	-0.05971	0.034415	-1.72885	0.085022142	-0.1271609	0.007745
Neotyphloceras chilensis	-1.14817	0.853581	-1.39814	0.20423313	-2.82115	0.524823	-0.00524	0.012791	-0.39598	0.663328871	-0.0303125	0.019826
Neotyphloceras crassispina	-0.3701	0.708024	-0.52731	0.61199861	-1.7578	1.017598	-0.0059	0.010693	-0.51738	0.444059583	-0.0268559	0.015058
Neotyphloceras pardinasi	-0.22787	1.163466	-0.36699	0.50878323	-2.50822	2.052483	-0.04298	0.02553	-1.69396	0.097995173	-0.0930222	0.007053
Nosopsyllus fasciatus	-1.54834	1.040573	-1.54791	0.1792187	-3.58783	0.491147	-0.00624	0.015438	-0.38966	0.618447063	-0.0364961	0.024022
Plocopsylla lewisi	0.733773	1.479962	0.232932	0.53308975	-2.1669	3.634445	-0.06671	0.038362	-1.79053	0.07611539	-0.141898	0.008478
Plocopsylla reigi	1.360517	2.71042	0.33526	0.60927586	-3.95181	6.672843	-0.09448	0.078526	-1.73695	0.131620662	-0.2483865	0.059431
Plocopsylla viracocha	0.913326	1.483327	0.481052	0.65129427	-1.99394	3.820593	-0.07706	0.0433	-1.81628	0.072269691	-0.1619261	0.007805
Plocopsylla wolffsohni	-1.05434	0.936777	-1.12257	0.33282123	-2.89039	0.781714	-0.01757	0.016335	-1.04704	0.198254185	-0.0495881	0.014444
Pulex irritans	-0.2908	2.009503	0.130065	0.34592824	-4.22935	3.647753	-0.03177	0.027663	-1.28591	0.103484125	-0.0859923	0.022444
Sphinctopsylla ares	0.718124	0.735848	1.048594	0.11198997	-0.72411	2.160359	-0.02797	0.012872	-2.08048	0.016899147	-0.0532016	-0.00274
Tetrapsyllus amplus	4.447185	9.069987	-0.14199	0.66874092	-13.3297	22.22403	-0.18185	0.247913	-1.4877	0.193438007	-0.6677522	0.304049
Tetrapsyllus corfidii	-0.92542	0.801177	-1.09383	0.45714912	-2.4957	0.644855	-0.01164	0.012802	-0.96038	0.206908552	-0.0367298	0.013452
Tetrapsyllus rhombus	-0.1263	0.823548	-0.14824	0.57745682	-1.74042	1.487826	-0.01525	0.012874	-1.16127	0.089657178	-0.0404868	0.009979
Tetrapsyllus satyrus	-1.64571	0.94749	-1.72607	0.08769451	-3.50276	0.211336	-0.01385	0.016617	-0.86964	0.411885411	-0.0464218	0.018715
Tetrapsyllus tantillus	-0.84707	0.756768	-1.14473	0.27544031	-2.33031	0.636168	-0.00647	0.011724	-0.52986	0.609371161	-0.0294474	0.016511
Overall	-0.76927	0.133687	-5.75343	3.41E-06	-1.03129	-0.50725	-0.02132	0.002463	-8.63709	1.06E-10	-0.0261509	-0.0165

Hantavirus	1.091335	1.741994	-0.07489	0.4105514	-2.32291	4.505581	-0.04736	0.029526	-1.61738	0.107183852	-0.1052278	0.010513
Overall including hantavirus	-0.75944	0.131915	-5.75665	3.69E-06	-1.01799	-0.50089	-0.02145	0.002433	-8.79762	2.38E-09	-0.0262161	-0.01668
